# Baseline and 72-h dynamic laboratory deficit burden for mortality through day 90 after acute ischemic stroke in critical care: landmark analyses with temporal validation

**DOI:** 10.3389/fneur.2026.1886148

**Published:** 2026-09-03

**Authors:** Guangwei Gu, Haonan Shi, Guofang Wang, Juluo Chen

**Affiliations:** 1 Fuyang People’s Hospital, Bengbu Medical University Affiliated Fuyang Hospital, Fuyang, Anhui, China; 2 Jiangxi Provincial People’s Hospital, First Affiliated Hospital of Nanchang Medical College, Nanchang, Jiangxi, China

**Keywords:** acute ischemic stroke, critical care, FI-Lab, laboratory deficit accumulation, landmark analysis, MIMIC-IV, mortality

## Abstract

**Background:**

A laboratory-based frailty index (FI-Lab) summarizes routinely measured deficits, but during critical illness it may reflect acute derangement and underlying vulnerability. Its baseline and dynamic roles require prediction times that precede outcome follow-up.

**Methods:**

Using MIMIC-IV v3.1, we performed internal temporal validation with separate 24-h and 72-h landmarks. The 24-h cohort comprised patients alive with evaluable 0–24 h FI-Lab; the 72-h cohort comprised those alive with evaluable baseline and 24–72 h FI-Lab. Each outcome was death after the applicable landmark through day 90 after ICU admission. Clinical logistic models were compared with models incorporating baseline FI-Lab, change direction, a four-level phenotype, or continuous baseline and change. We assessed discrimination, calibration, decision curves, and paired bootstrap differences, with sensitivity analyses defined for the revised analysis.

**Results:**

Among 3,376 admissions, 39 deaths occurred by 24 h and 148 by 72 h. The complete-case 24-h cohort comprised 3,186 patients (development: *n* = 1,890, 559 events; temporal validation: *n* = 1,296, 339 events). Adding baseline FI-Lab improved validation AUC from 0.774 to 0.808 (paired difference 0.034, 95% CI 0.022–0.047; *p* < 0.001) and reduced the Brier score from 0.162 to 0.152 (difference −0.011, 95% CI −0.015 to −0.006; *p* < 0.001). The complete-case 72-h cohort comprised 2,918 patients (development: *n* = 1,754, 473 events; validation: *n* = 1,164, 287 events). Baseline FI-Lab improved validation AUC from 0.752 to 0.791. Adding change direction or continuous change yielded AUCs of 0.797 and 0.798, respectively, but neither improvement over baseline FI-Lab was statistically supported; the four-level phenotype had AUC 0.767.

**Conclusion:**

Baseline FI-Lab improved internally validated prediction of mortality through day 90 among patients alive and evaluable at 24 h. At 72 h, dynamic change added limited global predictive information, and the phenotype remained exploratory. External validation is required before clinical use.

## 1 Introduction

Stroke remains a major source of death and long-term disability worldwide, and the contemporary burden remains substantial despite progress in prevention and acute treatment (1, 2). In acute ischemic stroke, the expansion of mechanical thrombectomy eligibility has changed the therapeutic landscape, especially for patients selected by clinical-imaging mismatch and perfusion imaging (3, 4). These advances have made acute management more precise, but they have not eliminated the need for early risk stratification in patients who require critical care.

Risk after acute ischemic stroke is not determined by the infarct alone. Patients admitted to the ICU often have respiratory failure, hemodynamic instability, infection, renal dysfunction, malignancy, or other competing systemic burdens. Frailty-related vulnerability is therefore relevant to stroke prognosis. Recent studies have linked brain frailty and neuroimaging markers of frailty to outcomes after thrombolysis and thrombectomy (5–7). However, a laboratory deficit index measured during critical illness cannot be assumed to represent premorbid frailty alone, because acute organ dysfunction, treatment, and monitoring intensity also shape its value.

MIMIC-IV provides an appropriate platform for this question because it contains granular critical care data, repeated laboratory measurements, ICU interventions, and mortality information (8). Existing MIMIC-IV studies in acute ischemic stroke have examined machine-learning mortality prediction, systemic immune-inflammation index, pan-immune-inflammation value, lactate dehydrogenase, and static FI-Lab in selected stroke subgroups (9–14). In parallel, a recent endovascular therapy cohort showed that FI-Lab can predict mortality in elderly patients with large vessel occlusion (15). These studies confirm the prognostic relevance of laboratory burden, but most treat laboratory information as a baseline exposure.

FI-Lab is attractive for ICU research because it uses routinely measured tests and operationalizes deficit accumulation without requiring functional questionnaires. It has been associated with mortality in critical acute myocardial infarction, acute pancreatitis, mechanically ventilated critical illness, and older hospitalized adults (16–19). Population studies further support frailty indices as markers of biological vulnerability (20–22), while longitudinal cardiovascular studies show that changes in frailty-related burden may add information beyond a single measurement (23–26). In the ICU, however, a valid dynamic analysis must account for the fact that follow-up measurements are available only after patients have survived long enough to be retested.

Here, we evaluated baseline and early dynamic FI-Lab in critically ill patients with acute ischemic stroke using explicit 24-h and 72-h landmark analyses. We hypothesized that baseline FI-Lab would add predictive information to clinical variables, while early change and the dynamic phenotype would serve primarily as secondary descriptions of laboratory trajectories. We also examined robustness to alternative phenotype cutoffs, matched laboratory domains, dynamic evaluability, early discharge, overlap with chronic kidney disease and diabetes, and mechanical ventilation.

## 2 Materials and methods

### 2.1 Study design and data source

We performed a retrospective cohort study using MIMIC-IV v3.1, a deidentified electronic health record database from Beth Israel Deaconess Medical Center (8). Reporting followed TRIPOD principles (27). Data extraction used Google BigQuery Standard SQL. A separate Reproducibility Package accompanies this revision and contains the complete cohort query, FI-Lab construction and landmark-analysis scripts, final table and figure code, quality-control records, and the aggregate Step 4 output archive. Credentialed users can regenerate the restricted patient-level analytic files locally; no MIMIC-derived patient-level data are redistributed.

### 2.2 Study population

Adult ICU patients were identified from all diagnosis positions using ICD-10-CM I63.x or ICD-9-CM 433x1, 434x1, or 436. Admissions containing subarachnoid hemorrhage, intracerebral or other nontraumatic intracranial hemorrhage, or transient ischemic attack codes (ICD-10-CM I60-I62 or G45; ICD-9-CM 430–432 or 435) were excluded. Prior-stroke or sequela codes neither established eligibility by themselves nor excluded a concurrent acute ischemic stroke. Only the first eligible ICU stay per patient was retained. Patients younger than 18 years, those with inconsistent timing, or those without ascertainable vital status through day 90 were excluded. A qualifying AIS diagnosis at sequence position 1 defined principal-diagnosis AIS for the requested sensitivity analysis. Full extraction logic and identifiers are provided in Supplementary Methods and Reproducibility Package.

### 2.3 FI-Lab construction and dynamic phenotype

FI-Lab was adapted for this study as an acute laboratory deficit-accumulation measure rather than a validated measure of premorbid frailty. It comprised white blood cell count, hemoglobin, platelet count, red cell distribution width, creatinine, blood urea nitrogen, glucose, sodium, potassium, and bicarbonate. Each available value was coded as normal or abnormal using study-defined ranges (Supplementary Table S1), and FI-Lab equaled abnormal domains divided by evaluable domains. Baseline FI-Lab required at least six domains and used the earliest value in 0–24 h. When several follow-up values were present, the result closest to 48 h within 24–72 h was selected, with the earlier result used to resolve ties.

Follow-up FI-Lab required at least max(3, ceiling [0.60 × the number of baseline-evaluable domains]) repeated domains. Change was follow-up minus baseline FI-Lab. A decrease of at least 0.125 was classified as improvement, an increase of at least 0.125 as worsening, and smaller changes as stable. The 0.125 cutoff approximates a one-deficit shift when eight domains are observed; it is an analytic threshold, not a validated clinical boundary. High load was defined by the development-cohort median (0.30) and fixed before temporal validation. Four phenotypes were defined: low-load stable, low-load worsened, high-load improved, and high-load persistent. Sensitivity analyses crossed high-load cutoffs of 0.20, 0.30, and 0.40 with change cutoffs of 0.10, 0.125, and 0.15; they also used continuous baseline and change terms and scores restricted to at least six laboratory domains shared by both time points.

### 2.4 Outcome and covariates

For the revised analysis, two prediction times were defined. The 24-h landmark cohort comprised patients alive with evaluable baseline FI-Lab at 24 h; the 72-h baseline pool comprised patients alive with evaluable baseline FI-Lab, and the dynamic cohort additionally required evaluable follow-up FI-Lab at 72 h. The outcome for each model was all-cause death after its landmark through day 90 after ICU admission. Exact in-hospital death time was preferred. If death time was unavailable, a recorded death date on or before the landmark calendar date was classified as pre-landmark in the primary analysis. Because within-day ordering was unavailable for these date-only records, a sensitivity analysis instead treated all date-only deaths as alive at the landmark. Clinical covariates were age, sex, minimum GCS in the first 24 h, vasopressor use, mechanical ventilation, hypertension, diabetes, atrial fibrillation, ischemic heart disease, heart failure, chronic kidney disease, chronic obstructive pulmonary disease, and malignancy. Minimum GCS was treated as a pragmatic consciousness/severity marker that may be influenced by sedation and intubation. No multiple imputation was performed: clinical models used complete cases, while partial laboratory panels were handled only through the stated FI-Lab evaluability rules.

### 2.5 Model development and validation

Anchor-year groups 2008–2016 formed the development cohort and 2017–2022 the temporal-validation cohort. At the 24-h landmark, Model A contained the 13 clinical predictors and Model B added baseline FI-Lab. At the 72-h landmark, Models A and B were refitted in the dynamic cohort; Model C added categorical change direction to Model B, Model D replaced separate FI-Lab terms with the four-level dynamic phenotype, and Model E entered baseline FI-Lab and continuous change simultaneously. Continuous predictors were entered linearly; sex, treatments, and comorbidities were binary; stable change and low-load stable phenotype were reference categories. Models were fitted only in development data and applied without refitting to temporal validation data. The temporal split was considered internal temporal validation within one healthcare system.

### 2.6 Statistical analysis and model performance

Discrimination was summarized by AUC with DeLong 95% confidence intervals. Paired, outcome-stratified bootstrap resampling with 2,000 replicates quantified differences in AUC and Brier score in temporal validation. Calibration was evaluated using Brier score, calibration intercept, calibration slope, and calibration plots. Decision curves covered threshold probabilities from 0.05 to 0.50 (28). Sample-size adequacy was considered in relation to the number of outcome events and model complexity, with reference to current guidance for clinical prediction model development (29). Variance inflation factors assessed collinearity. Development and temporal-validation cohorts, and dynamically evaluable and non-evaluable patients, were compared using standardized mean differences. In the development cohort, natural cubic splines with restricted linear tails (4 degrees of freedom) were compared with linear terms by likelihood-ratio tests: baseline FI-Lab was assessed at 24 h, and baseline FI-Lab and FI-Lab change were assessed at 72 h with mutual adjustment. Sensitivity analyses addressed date-only death classification, cutoff choice, laboratory-panel mismatch, early discharge, chronic kidney disease/diabetes overlap, mechanical ventilation, and restriction to principal-diagnosis AIS. Analyses used R 4.5.3, data.table 1.18.2.1, pROC 1.19.0.1, and random seed 20260723.

## 3 Results

### 3.1 Cohort construction

**Figure 1:**
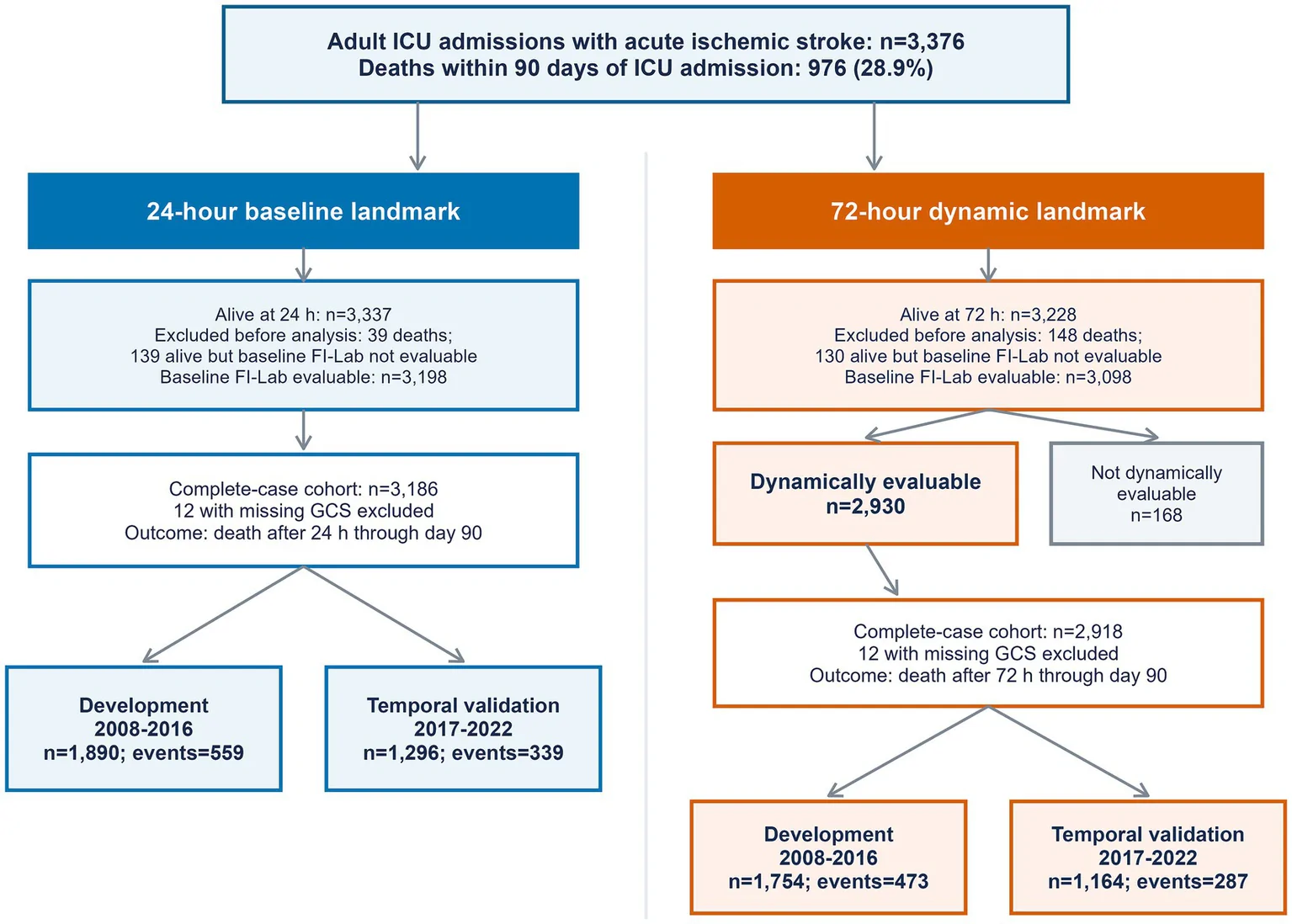
Study flow diagram. The diagram separately reports the 24-h baseline and 72-h dynamic landmark pathways. At 24 h it shows 39 pre-landmark deaths, 139 patients alive but without evaluable baseline FI-Lab, and 12 complete-case exclusions for missing GCS. At 72 h it shows 148 pre-landmark deaths, 130 patients alive but without evaluable baseline FI-Lab, 168 patients without evaluable dynamic FI-Lab, and 12 complete-case exclusions for missing GCS, followed by the temporal splits and post-landmark events.

The extraction identified 3,376 adult ICU admissions, of whom 976 (28.9%) died through day 90 after ICU admission. Thirty-nine deaths occurred by 24 h, including 38 identified by exact timestamps and one by date only; 148 occurred by 72 h, including 134 exact-timestamp and 14 date-only deaths. At 24 h, 3,337 patients were alive; 139 were alive but lacked an evaluable baseline FI-Lab, leaving 3,198 alive with evaluable baseline FI-Lab. Twelve with missing GCS were excluded, producing 3,186 complete cases (development: *n* = 1,890, 559 post-landmark deaths; temporal validation: *n* = 1,296, 339 deaths). At 72 h, 3,228 patients were alive; 130 were alive but lacked an evaluable baseline FI-Lab, leaving 3,098 in the baseline pool. Of these, 2,930 were dynamically evaluable and 168 were not. Excluding 12 dynamically evaluable patients with missing GCS left 2,918 complete cases (development: *n* = 1,754, 473 post-landmark deaths; validation: *n* = 1,164, 287 deaths; Figure 1).

### 3.2 Baseline characteristics across dynamic FI-Lab phenotypes

Dynamic evaluability was associated with the clinical course (Supplementary Table S9). Compared with 2,930 dynamically evaluable patients, the 168 non-evaluable patients were more often discharged from hospital by 72 h (62.5% vs. 9.5%) or from the ICU by 72 h (90.5% vs. 45.3%), had higher minimum GCS, and were less often mechanically ventilated (5.4% vs. 36.4%) or treated with vasopressors (3.6% vs. 22.8%). Post-landmark mortality was 22.6 and 26.1%, respectively. Development-versus-validation comparisons are reported for the full admission cohort and both complete-case landmark cohorts in Supplementary Table S21. The largest absolute standardized mean difference was for chronic kidney disease (0.215 in the full cohort, 0.206 at 24 h, and 0.209 at 72 h). Within the complete-case dynamic cohort, phenotype sample sizes were 931 low-load stable, 207 low-load worsened, 356 high-load improved, and 1,424 high-load persistent patients; the persistent group was older and had greater neurological, organ-support, and comorbidity burden (Table 1).

**Table 1:** Characteristics of the 72-h complete-case cohort according to dynamic FI-Lab phenotype.

characteristic	Low-load stable (*n* = 931)	Low-load worsened (*n* = 207)	High-load improved (*n* = 356)	High-load persistent (*n* = 1,424)
Age, years	66.5 (15.3)	69.5 (15.6)	69.4 (15.0)	70.4 (15.0)
Male sex	503/931 (54.0%)	96/207 (46.4%)	181/356 (50.8%)	714/1,424 (50.1%)
Minimum GCS in first 24 h	14.0 (11.0–15.0)	10.0 (6.0–14.0)	11.0 (5.8–14.0)	9.0 (3.0–14.0)
Vasopressor use in first 24 h	83/931 (8.9%)	44/207 (21.3%)	93/356 (26.1%)	447/1,424 (31.4%)
Mechanical ventilation in first 24 h	151/931 (16.2%)	82/207 (39.6%)	153/356 (43.0%)	680/1,424 (47.8%)
Hypertension	668/931 (71.8%)	154/207 (74.4%)	278/356 (78.1%)	1,117/1,424 (78.4%)
Diabetes	210/931 (22.6%)	63/207 (30.4%)	150/356 (42.1%)	543/1,424 (38.1%)
Atrial fibrillation	269/931 (28.9%)	78/207 (37.7%)	138/356 (38.8%)	614/1,424 (43.1%)
Ischemic heart disease	203/931 (21.8%)	60/207 (29.0%)	129/356 (36.2%)	563/1,424 (39.5%)
Heart failure	119/931 (12.8%)	37/207 (17.9%)	108/356 (30.3%)	492/1,424 (34.6%)
Chronic kidney disease	37/931 (4.0%)	13/207 (6.3%)	96/356 (27.0%)	461/1,424 (32.4%)
COPD	63/931 (6.8%)	21/207 (10.1%)	37/356 (10.4%)	190/1,424 (13.3%)
Malignancy	62/931 (6.7%)	15/207 (7.2%)	54/356 (15.2%)	245/1,424 (17.2%)
Baseline FI-Lab	0.13 (0.07)	0.12 (0.08)	0.55 (0.18)	0.45 (0.14)
FI-Lab change	0.00 (0.08)	0.24 (0.07)	−0.24 (0.08)	0.03 (0.12)
Post-landmark mortality	86/931 (9.2%)	44/207 (21.3%)	101/356 (28.4%)	529/1,424 (37.1%)

Age, baseline FI-Lab, and FI-Lab change are mean (SD); minimum GCS is median (interquartile range); categorical variables are *n*/*N* (%). Mortality denotes death after the 72-h landmark through day 90 after ICU admission. FI-Lab, laboratory-based frailty index; GCS, Glasgow Coma Scale; COPD, chronic obstructive pulmonary disease.

### 3.3 Mortality gradient by dynamic phenotype

**Figure 2:**
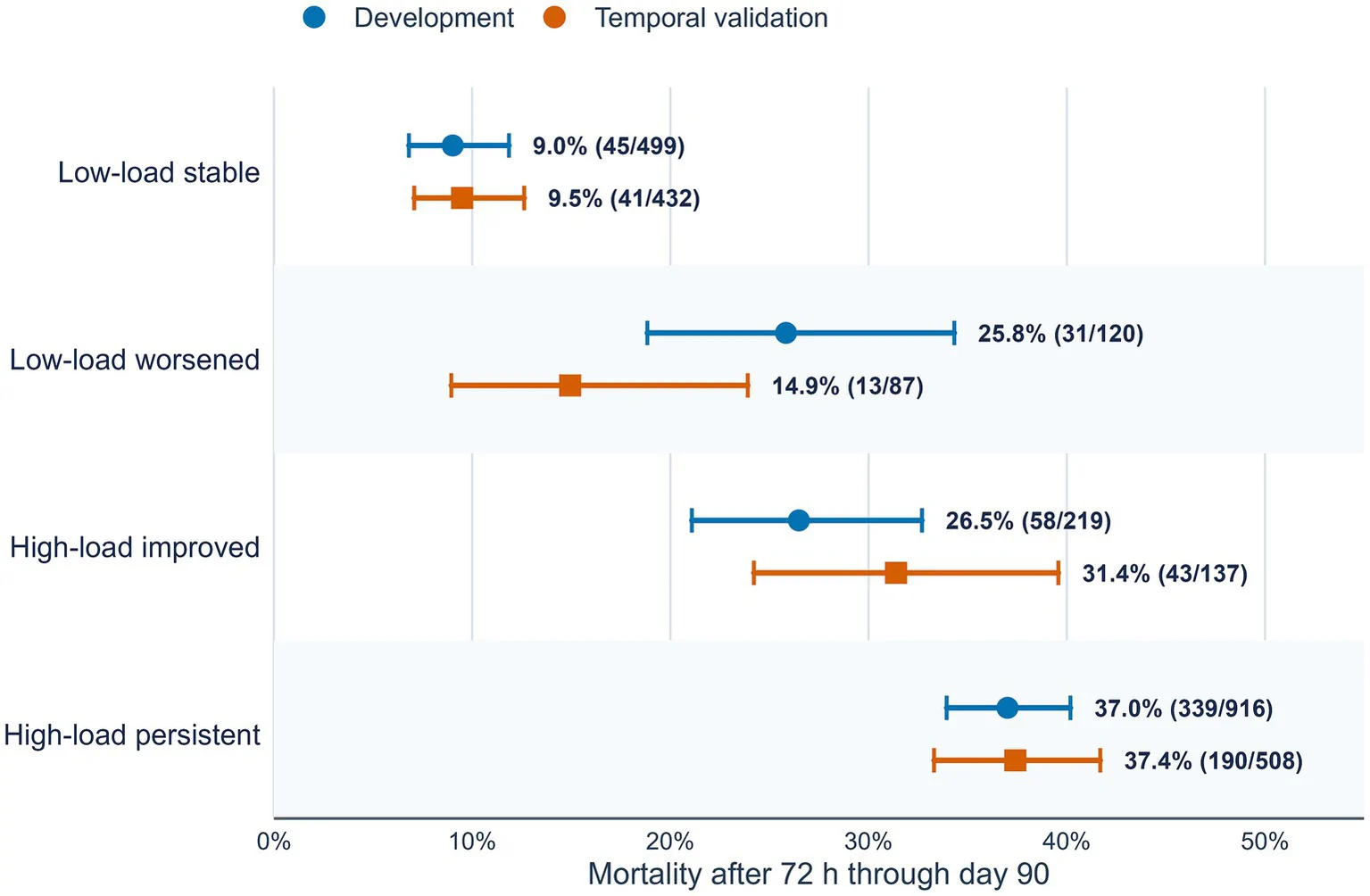
Post-landmark mortality through day 90 across 72-h dynamic FI-Lab phenotypes. Points show observed proportions with exact 95% confidence intervals in development and temporal-validation cohorts. The extreme groups were quantitatively consistent; intermediate groups are presented descriptively because of smaller samples and between-cohort variation in absolute risk.

Observed post-landmark mortality increased across the four phenotypes in both temporal cohorts (Figure 2). Development-cohort mortality was 9.0% (45/499) for low-load stable, 25.8% (31/120) for low-load worsened, 26.5% (58/219) for high-load improved, and 37.0% (339/916) for high-load persistent patients. Corresponding validation rates were 9.5% (41/432), 14.9% (13/87), 31.4% (43/137), and 37.4% (190/508). The extreme groups were quantitatively consistent, whereas the smaller low-load worsened group showed wider uncertainty and between-cohort variation (Supplementary Table S13).

### 3.4 Regression results for FI-Lab metrics

At the 24-h landmark, each 0.1 increase in baseline FI-Lab was associated with higher post-landmark mortality in Model B (OR 1.32, 95% CI 1.23–1.41; *p* < 0.001). At the 72-h landmark, Model C estimated an OR of 0.43 (95% CI 0.29–0.63; *p* < 0.001) for improved versus stable change and 1.54 (95% CI 1.10–2.16; *p* = 0.012) for worsened versus stable change, conditional on baseline FI-Lab and clinical covariates. In Model D, relative to low-load stable, adjusted ORs were 2.45 (95% CI 1.42–4.21; *p* = 0.001) for low-load worsened, 2.09 (95% CI 1.31–3.32; *p* = 0.002) for high-load improved, and 3.14 (95% CI 2.17–4.53; *p* < 0.001) for high-load persistent phenotypes (Table 2). In Model E, the ORs per 0.1 increase were 1.48 (95% CI 1.37–1.61; *p* < 0.001) for baseline FI-Lab and 1.33 (95% CI 1.21–1.45; *p* < 0.001) for change.

**Table 2:** Multivariable associations in the 72-h dynamic phenotype model.

Predictor	Adjusted OR (95% CI)	*p* value
Age, per year	1.04 (1.03–1.05)	<0.001
Male sex	1.19 (0.94–1.52)	0.152
Minimum GCS, per point	0.90 (0.86–0.94)	<0.001
Vasopressor use	0.95 (0.70–1.28)	0.734
Mechanical ventilation	1.10 (0.73–1.65)	0.659
Hypertension	0.75 (0.55–1.02)	0.068
Diabetes	0.99 (0.77–1.28)	0.941
Atrial fibrillation	0.83 (0.65–1.07)	0.160
Ischemic heart disease	1.27 (0.98–1.64)	0.067
Heart failure	1.21 (0.91–1.59)	0.186
Chronic kidney disease	1.65 (1.24–2.20)	<0.001
COPD	1.22 (0.87–1.72)	0.252
Malignancy	2.49 (1.82–3.42)	<0.001
Low-load worsened	2.45 (1.42–4.21)	0.001
High-load improved	2.09 (1.31–3.32)	0.002
High-load persistent	3.14 (2.17–4.53)	<0.001

Odds ratios were estimated in the development cohort (*n* = 1,754; 473 post-landmark deaths) with simultaneous adjustment for all listed predictors. Low-load stable is the reference phenotype. Associations are not causal.

### 3.5 Model performance

**Figure 3:**
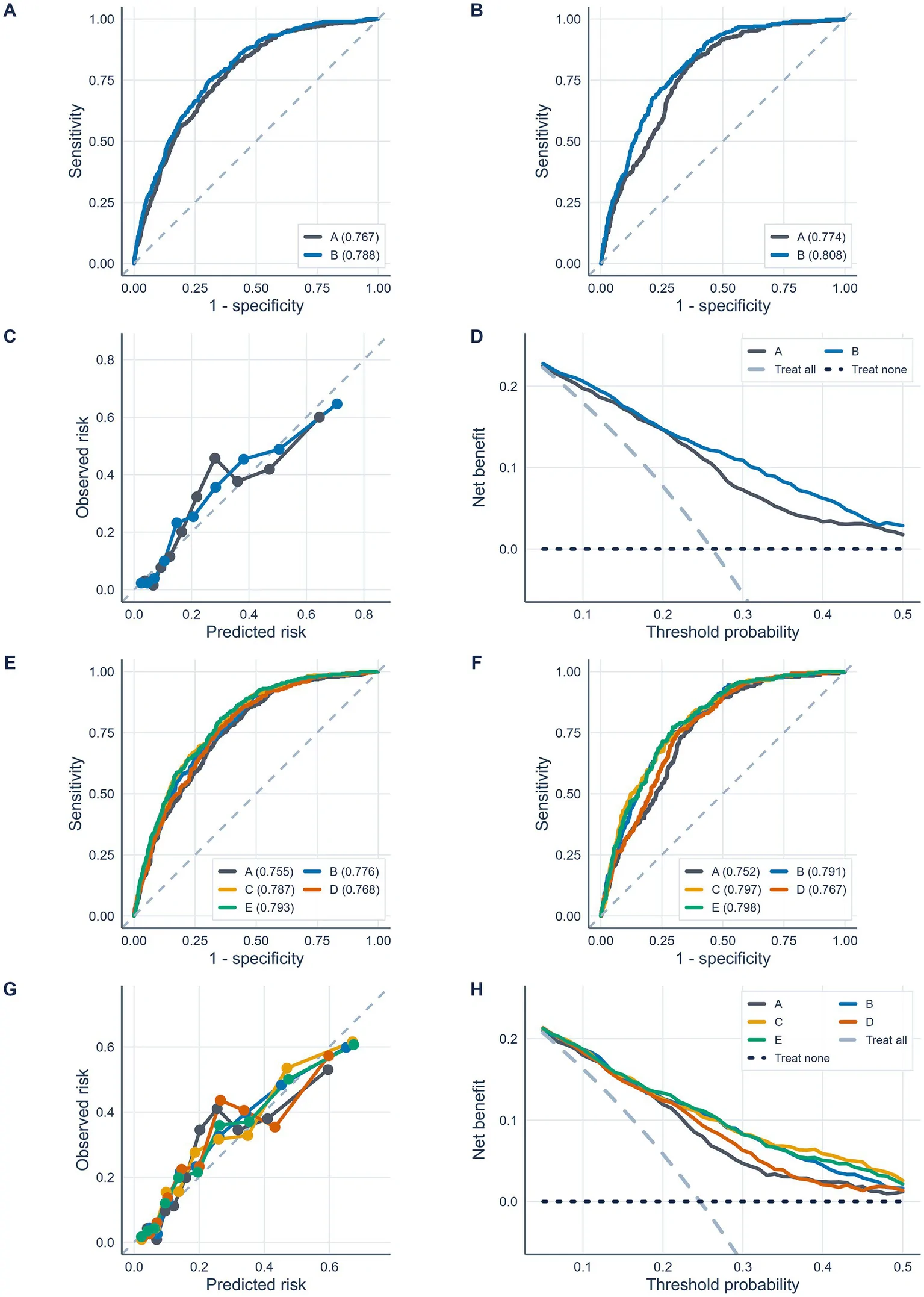
Model performance comparison at the 24-h and 72-h landmarks. (**A,B,E,F**) Receiver operating characteristic curves comparing prediction models. (**C,G**) Calibration plots showing agreement between predicted and observed risks. (**D,H**) Decision curve analysis showing clinical net benefit across threshold probabilities. Panels **A–D** represent the 24-h landmark analysis, and panels **E–H** represent the 72-h dynamic landmark analysis.

Model performance is summarized in Table 3 and Figure 3. In 24-h temporal validation, Model B improved AUC from 0.774 (95% CI 0.747–0.801) to 0.808 (95% CI 0.784–0.833), with a paired difference of 0.034 (95% CI 0.022–0.047; *p* < 0.001). The Brier score improved from 0.162 to 0.152 (difference −0.011, 95% CI −0.015 to −0.006; *p* < 0.001); the calibration intercept and slope for Model B were 0.095 and 0.943. In 72-h validation, AUCs were 0.752 for Model A, 0.791 for Model B, 0.797 for Model C, 0.767 for Model D, and 0.798 for Model E. Model B improved AUC by 0.039 versus Model A (95% CI 0.025–0.054; *p* < 0.001) and produced a paired Brier difference of −0.011 (95% CI −0.015 to −0.007; *p* < 0.001). Relative to Model B, paired AUC differences were 0.006 for Model C (*p* = 0.184), −0.024 for Model D (*p* < 0.001), and 0.006 for Model E (*p* = 0.192; Supplementary Table S7).

**Table 3:** Model performance in development and temporal-validation cohorts at each landmark.

Landmark	Cohort	Model	Events/*n*	AUC (95% CI)	Brier	Calibration intercept	Calibration slope
24 h	Development	Model A	559/1,890	0.767 (0.745–0.790)	0.171	0.000	1.000
24 h	Development	Model B	559/1,890	0.788 (0.766–0.809)	0.165	0.000	1.000
24 h	Temporal validation	Model A	339/1,296	0.774 (0.747–0.801)	0.162	0.098	0.941
24 h	Temporal validation	Model B	339/1,296	0.808 (0.784–0.833)	0.152	0.095	0.943
72 h	Development	Model A	473/1,754	0.755 (0.731–0.779)	0.167	0.000	1.000
72 h	Development	Model B	473/1,754	0.776 (0.753–0.799)	0.161	0.000	1.000
72 h	Development	Model C	473/1,754	0.787 (0.765–0.810)	0.158	0.000	1.000
72 h	Development	Model D	473/1,754	0.768 (0.745–0.792)	0.163	0.000	1.000
72 h	Development	Model E	473/1,754	0.793 (0.771–0.815)	0.157	0.000	1.000
72 h	Temporal validation	Model A	287/1,164	0.752 (0.722–0.782)	0.163	0.129	0.916
72 h	Temporal validation	Model B	287/1,164	0.791 (0.763–0.819)	0.152	0.120	0.938
72 h	Temporal validation	Model C	287/1,164	0.797 (0.769–0.825)	0.150	0.114	0.910
72 h	Temporal validation	Model D	287/1,164	0.767 (0.738–0.795)	0.159	0.161	0.902
72 h	Temporal validation	Model E	287/1,164	0.798 (0.770–0.825)	0.150	0.104	0.906

AUC, area under the receiver operating characteristic curve. Models were fitted in development and applied without refitting to temporal validation. Model A, clinical reference; Model B, clinical plus baseline FI-Lab; Model C, Model B plus change direction; Model D, clinical plus four-level dynamic phenotype; Model E, clinical plus continuous baseline FI-Lab and continuous change. Events are deaths after the relevant landmark through day 90 after ICU admission.

### 3.6 Sensitivity analyses

**Figure 4:**
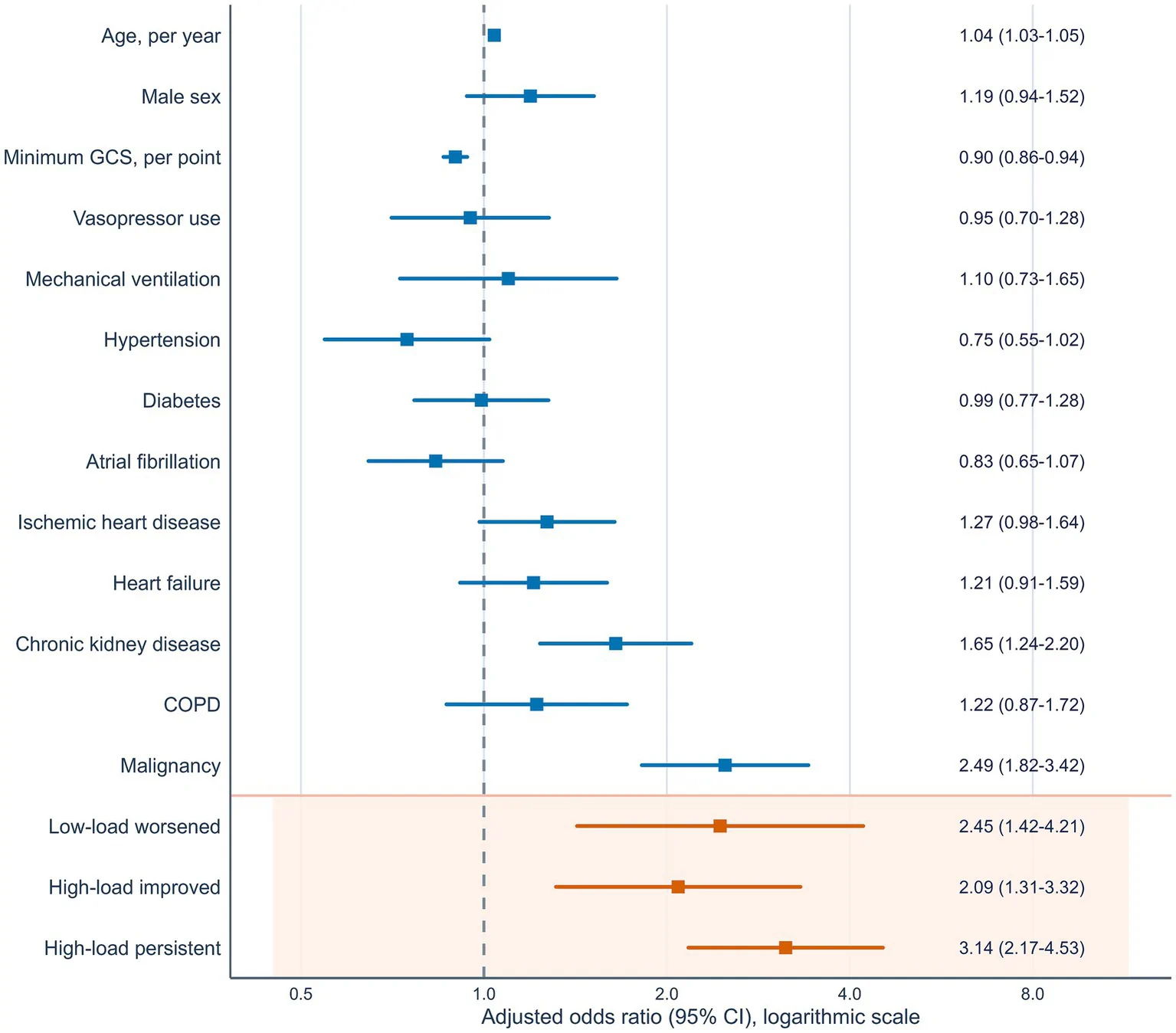
Adjusted associations in the 72-h dynamic phenotype logistic model. Odds ratios and 95% confidence intervals are displayed on a logarithmic scale; low-load stable is the reference phenotype. Coefficients are associational and should not be interpreted causally.

Sensitivity analyses supported the main interpretation (Supplementary Tables S10–S23 and Supplementary Figure S1). Across nine cutoff combinations, 72-h validation AUC ranged from 0.749 to 0.776. Restricting change calculations to at least six laboratory domains available at both time points yielded AUC 0.767 and Brier score 0.159. Primary and matched-domain changes were highly correlated (Pearson *r* = 0.995); phenotype agreement was 99.5%, with Cohen kappa 0.992 (Supplementary Tables S11B,C). The primary analysis excluded one date-only death from the 24-h risk set and 14 from the 72-h risk set. Treating all date-only deaths as alive at the landmark allowed one additional patient into the 24-h development analysis and two into the 72-h analyses; validation AUC and Brier score changed by less than 0.001 (Supplementary Table S20). Natural cubic splines showed no evidence of nonlinearity for baseline FI-Lab at 24 h (*p* = 0.210), baseline FI-Lab adjusted for change at 72 h (*p* = 0.277), or FI-Lab change adjusted for baseline at 72 h (*p* = 0.206). AIS was the principal diagnosis in 1,964/3,376 admissions (58.2%); principal-diagnosis-only analyses preserved discrimination and the relative model pattern (Supplementary Tables S22A,B). The 24-h baseline model was essentially unchanged after removing chronic kidney disease and diabetes covariates (AUC 0.810) and retained discrimination among patients not mechanically ventilated (AUC 0.848). The maximum conventional VIF was 3.45 and the maximum adjusted GVIF was 1.86 (Figure 4).

## 4 Discussion

This study provides two time-aligned findings. First, baseline FI-Lab improved mortality prediction among patients alive and evaluable at the 24-h prediction point, with statistically supported gains in validation AUC and prediction error. Second, among patients alive and dynamically evaluable at 72 h, both categorical and continuous change carried adjusted prognostic information, but neither produced a statistically supported increase in validation AUC beyond baseline FI-Lab. The four-level phenotype performed worse than the baseline FI-Lab model in global prediction and should be interpreted as exploratory risk stratification.

The most reproducible phenotype information lay at the extremes. Low-load stable mortality was 9.0–9.5% and high-load persistent mortality 37.0–37.4% across the two cohorts. The intermediate categories appeared in the same order, but their absolute estimates were less stable: low-load worsened mortality was 25.8% in development and 14.9% in validation, with only 120 and 87 patients. We therefore do not regard all four categories as validated ordinal risk states. The phenotype is better viewed as a clinically readable description of laboratory trajectories that may support hypothesis generation and subgroup characterization.

The landmark structure changes the scope of inference. Admission-cohort mortality through day 90 was 28.9%, whereas each model counted only deaths after its prediction time; 39 patients died by 24 h and 148 by 72 h under the primary best-available timing rule. The baseline result applies to patients alive and evaluable at 24 h, and the dynamic result applies to the more selected group alive and retested at 72 h. Dynamic non-evaluability was strongly related to early discharge and lower treatment intensity, confirming that repeated testing was not random. The alternative date-only classification produced virtually unchanged performance, but it cannot resolve the actual within-day order for those records. The results should not be extrapolated to patients who die before the landmark or lack the required measurements.

These results complement rather than duplicate prior MIMIC-IV stroke studies. Earlier work has developed machine-learning mortality models or examined single inflammatory and laboratory markers such as systemic immune-inflammation index, pan-immune-inflammation value, and lactate dehydrogenase in critically ill stroke populations (9–13). Such studies demonstrate that systemic inflammation and laboratory abnormalities are prognostically relevant, but single markers tend to represent narrow biological pathways. Static FI-Lab studies in ischemic stroke with carotid stenosis and preprocedural FI-Lab studies in endovascular therapy have already shown that laboratory deficit accumulation is associated with stroke outcomes (14, 15). The present analysis extends this line of work by focusing on a 72-h ICU window and by separating static prediction performance from dynamic risk stratification.

The findings fit the broader frailty literature, but terminology requires caution. FI-Lab has predicted mortality in several critical-care and hospital populations (16–19), while population cohorts link deficit accumulation to biological vulnerability (20–22). In this study, however, the ten-domain score was adapted to available ICU laboratories and captures acute inflammation, cytopenia, kidney dysfunction, metabolic disturbance, and electrolyte or acid–base derangement. It is therefore more accurately interpreted as acute laboratory deficit burden that may contain, but does not isolate, premorbid frailty or physiological reserve.

A practical interpretation is that baseline FI-Lab is the principal predictive marker, whereas 72-h change summarizes the early course among patients who survive long enough to be reassessed. Model C and the continuous Model E were numerically stronger than Model B, but their paired AUC gains were only 0.006 and confidence intervals included no improvement. Categorizing baseline and change into the phenotype also discarded information and reduced discrimination. These findings support retaining continuous FI-Lab when prediction is the goal and using phenotypes cautiously for descriptive communication.

The baseline model also showed favorable validation calibration and prediction error. Its 24-h decision curve exceeded the clinical model throughout the assessed threshold range, but decision-curve superiority in a retrospective internal validation does not establish a treatment rule. The models require evaluation in other institutions, contemporary care pathways, and clinically defined decision contexts before any threshold can be recommended.

Several adjusted findings require noncausal interpretation. FI-Lab overlaps conceptually with diabetes and chronic kidney disease through glucose and creatinine, although collinearity was limited and discrimination was unchanged when those covariates were removed. Hypertension had an inverse but nonsignificant coefficient in the phenotype model (OR 0.75, 95% CI 0.55–1.02; *p* = 0.068) and was also nonsignificant in the 24-h baseline-FI-Lab model; this pattern may reflect coding, selection, treatment, or residual confounding rather than protection.

Several limitations should be acknowledged. This was a retrospective single-center database study with residual confounding. Landmarking prevents use of future information but restricts inference to survivors and does not remove selection from nonrandom laboratory retesting. FI-Lab was adapted from routinely available domains and may predominantly reflect acute physiological derangement; its data-derived 0.30 threshold and analytic change cutoff lack established clinical meaning. Although matched-domain and alternative-cutoff analyses were reassuring, the smaller intermediate phenotype groups remained quantitatively less stable. Minimum GCS is not stroke-specific and may reflect sedation, while structured data incompletely capture NIHSS, imaging severity, reperfusion, stroke subtype, and treatment timing. AIS was identified from diagnosis codes rather than clinical adjudication; 58.2% had AIS at diagnosis sequence position 1, and the principal-diagnosis-only sensitivity cannot remove coding misclassification. The outcome was all-cause rather than stroke-specific mortality, and date-only records retain unavoidable within-day timing uncertainty. Modest temporal differences in several baseline variables also indicate case-mix drift. Finally, the temporal split remains internal validation; external and prospective evaluation is required.

The study nevertheless has several strengths: explicit 24-h and 72-h prediction times, exclusion of pre-landmark deaths from the corresponding outcomes, a larger baseline-evaluable cohort for the baseline question, temporal rather than random validation, paired performance comparisons, transparent complete-case handling, direct assessment of dynamic evaluability, development-versus-validation standardized differences, and sensitivity analyses addressing date-only timing, cutoff choice, matched laboratory domains, early discharge, covariate overlap, mechanical ventilation, principal diagnosis, and functional form. Complete extraction and analysis code and aggregate outputs are supplied separately.

## 5 Conclusion

Among critically ill patients with acute ischemic stroke who were alive and evaluable at 24 h, baseline FI-Lab improved internally validated prediction of mortality through day 90. Among those alive and dynamically evaluable at 72 h, change measures added limited global predictive information and the four-level phenotype did not outperform baseline FI-Lab. The findings support cautious, time-specific risk stratification and require external validation before clinical use.

## Data Availability

The data analyzed in this study are available through PhysioNet after completion of the required credentialing process and data use agreement for MIMIC-IV v3.1. The analytic cohort was derived from structured hospital and ICU tables. Analysis code can be made available by the corresponding author on reasonable request, subject to MIMIC-IV data-use requirements.
