# Supplementary material for: Baseline and 72-h dynamic laboratory deficit burden for mortality through day 90 after acute ischemic stroke in critical care: landmark analyses with temporal validation
Source: Front Neurol. 2026 Sep 3;17:1886148. doi: 10.3389/fneur.2026.1886148 (PMC13583039; doi:10.3389/fneur.2026.1886148)
Supplement: Supplementary file 1 [file Supplementary_file_1.DOCX]

**Supplementary Material**

**Baseline and 72-Hour Dynamic Laboratory Deficit Burden for Mortality Through Day 90 After Acute Ischemic Stroke in Critical Care: Landmark Analyses With Temporal Validation**

**Supplementary Methods**

**Cohort identification and time origin.** MIMIC-IV v3.1 adult ICU stays were screened using all diagnosis positions. Acute ischemic stroke required ICD-10-CM I63.x or ICD-9-CM 433x1, 434x1, or 436; admissions with hemorrhagic stroke or transient ischemic attack codes were excluded. Prior-stroke and sequela codes neither established eligibility alone nor excluded a concurrent acute event. The first eligible ICU stay per patient was retained. Anchor-year groups 2008-2016 formed development and 2017-2022 internal temporal validation. A qualifying AIS code with diagnosis sequence number 1 defined principal-diagnosis AIS.

**FI-Lab extraction.** The ten-domain panel covered hematologic, renal, metabolic, and electrolyte/acid-base measures. Baseline used the earliest result in 0-24 h; follow-up used the result closest to 48 h within 24-72 h, resolving ties toward the earlier measurement. FI-Lab was the number of abnormal evaluable domains divided by the number evaluated. Baseline required at least six domains; follow-up required at least max(3, ceiling[0.60 × baseline-evaluable domains]).

**Landmarks, outcome, and missing data.** The 24-hour analysis included patients alive with evaluable baseline FI-Lab; the 72-hour analysis included patients alive with evaluable baseline and follow-up FI-Lab. Each model counted only deaths after the applicable landmark through day 90 after ICU admission. Exact in-hospital deathtime was used when available. Otherwise, a recorded death date on or before the landmark calendar date was classified as pre-landmark in the primary analysis. The alternative sensitivity analysis treated all date-only deaths as alive at the landmark. No imputation was performed. Clinical models used complete cases; partial laboratory panels were used only under the stated FI-Lab evaluability rules.

**Modeling and validation.** Logistic models were fitted in development and transferred without refitting to temporal validation. AUC confidence intervals used DeLong's method. Paired outcome-stratified bootstrap resampling (2,000 replicates) compared AUC and Brier score. Calibration intercepts/slopes, calibration plots, decision curves (thresholds 0.05-0.50), and collinearity diagnostics were reported. Development and temporal-validation cohorts were compared using standardized mean differences. In development data, natural cubic splines with restricted linear tails (4 degrees of freedom) assessed baseline FI-Lab at 24 h and mutually adjusted baseline FI-Lab and change at 72 h; likelihood-ratio tests compared spline and linear forms. Analyses used R 4.5.3, data.table 1.18.2.1, pROC 1.19.0.1, and random seed 20260723.

**Supplementary Table S1. FI-Lab laboratory thresholds.**

| **Laboratory domain** | **Unit** | **Reference range** | **Deficit definition** |
| --- | --- | --- | --- |
| White blood cell count | ×10⁹/L | 4.0-10.0 | <4.0 or >10.0 |
| Hemoglobin | g/dL | Male 13.0-17.5; female 12.0-16.0 | Outside sex-specific range |
| Platelet count | ×10⁹/L | 150-450 | <150 or >450 |
| Red cell distribution width | % | ≤14.5 | >14.5 |
| Creatinine | mg/dL | Male ≤1.3; female ≤1.1 | Above sex-specific upper limit |
| Blood urea nitrogen | mg/dL | 7-20 | <7 or >20 |
| Glucose | mg/dL | 70-180 | <70 or >180 |
| Sodium | mmol/L | 135-145 | <135 or >145 |
| Potassium | mmol/L | 3.5-5.0 | <3.5 or >5.0 |
| Bicarbonate | mmol/L | 22-29 | <22 or >29 |

*These ranges were defined in the executable analysis dictionary. FI-Lab is interpreted as acute laboratory deficit burden rather than a validated premorbid frailty scale.*

**Supplementary Table S2. Cohort and covariate extraction definitions.**

| **Construct** | **Source/window** | **Operational definition** |
| --- | --- | --- |
| Acute ischemic stroke | All diagnosis positions | ICD-10-CM I63.x; ICD-9-CM 433x1, 434x1, or 436 |
| Competing diagnosis exclusion | All diagnosis positions | ICD-10-CM I60-I62 or G45; ICD-9-CM 430-432 or 435 |
| Prior stroke/sequelae | Eligibility rule | Neither sufficient alone nor exclusionary with a concurrent acute ischemic stroke code |
| Index stay | ICU episode | First eligible ICU stay per patient |
| Baseline laboratories | 0-24 h | Earliest eligible value after ICU admission |
| Follow-up laboratories | 24-72 h | Value closest to 48 h; earlier value resolves ties |
| Minimum GCS | 0-24 h | Minimum documented total or complete eye+verbal+motor sum at a common chart time |
| Vasopressor use | 0-24 h | Any norepinephrine, epinephrine, phenylephrine, vasopressin, dopamine, or dobutamine record |
| Mechanical ventilation | 0-24 h | Ventilator, ventilation mode, tidal-volume, or PEEP charted event |
| Comorbidities | Hospital diagnoses | Defined ICD logic for hypertension, diabetes, AF, IHD, HF, CKD, COPD, and malignancy |
| Mortality | After landmark through ICU day 90 | Exact hospital deathtime preferred; patient DOD otherwise |

*Complete SQL logic and item identifiers are provided in the separate Reproducibility Package. Patient-level MIMIC-derived files are not redistributed.*

**Supplementary Table S3. Landmark cohort flow.**

| **item** | **n** |
| --- | --- |
| Full admission cohort | 3,376 |
| Deaths by 24 h (best available time) | 39 |
| Deaths by 72 h (best available time) | 148 |
| Alive and baseline FI-Lab evaluable at 24 h | 3,198 |
| Alive and baseline FI-Lab evaluable at 72 h | 3,098 |
| Dynamically evaluable at 72 h | 2,930 |
| Not dynamically evaluable among 72 h baseline pool | 168 |
| 24 h complete cases for clinical plus baseline FI-Lab | 3,186 |
| 72 h complete cases for all primary dynamic models | 2,918 |

**Supplementary Table S4. Complete model specifications.**

| **landmark** | **model** | **role** | **formula** |
| --- | --- | --- | --- |
| 24 h | Model_A | Clinical reference | outcome_24h ~ age_years + gender + gcs_min_24h + vasopressor_24h + ventilation_24h + comorb_hypertension + comorb_diabetes + comorb_atrial_fibrillation + comorb_ihd + comorb_heart_failure + comorb_ckd + comorb_copd + comorb_malignancy |
| 24 h | Model_B | Clinical plus baseline FI-Lab | outcome_24h ~ age_years + gender + gcs_min_24h + vasopressor_24h + ventilation_24h + comorb_hypertension + comorb_diabetes + comorb_atrial_fibrillation + comorb_ihd + comorb_heart_failure + comorb_ckd + comorb_copd + comorb_malignancy + b_filab_01 |
| 72 h | Model_A | Clinical reference | outcome_72h ~ age_years + gender + gcs_min_24h + vasopressor_24h + ventilation_24h + comorb_hypertension + comorb_diabetes + comorb_atrial_fibrillation + comorb_ihd + comorb_heart_failure + comorb_ckd + comorb_copd + comorb_malignancy |
| 72 h | Model_B | Clinical plus baseline FI-Lab | outcome_72h ~ age_years + gender + gcs_min_24h + vasopressor_24h + ventilation_24h + comorb_hypertension + comorb_diabetes + comorb_atrial_fibrillation + comorb_ihd + comorb_heart_failure + comorb_ckd + comorb_copd + comorb_malignancy + b_filab_01 |
| 72 h | Model_C | Clinical plus baseline FI-Lab plus change direction | outcome_72h ~ age_years + gender + gcs_min_24h + vasopressor_24h + ventilation_24h + comorb_hypertension + comorb_diabetes + comorb_atrial_fibrillation + comorb_ihd + comorb_heart_failure + comorb_ckd + comorb_copd + comorb_malignancy + b_filab_01 + change_dir |
| 72 h | Model_D | Clinical plus four-level dynamic phenotype | outcome_72h ~ age_years + gender + gcs_min_24h + vasopressor_24h + ventilation_24h + comorb_hypertension + comorb_diabetes + comorb_atrial_fibrillation + comorb_ihd + comorb_heart_failure + comorb_ckd + comorb_copd + comorb_malignancy + dynamic_phenotype |
| 72 h | Model_E | Clinical plus continuous baseline FI-Lab and continuous change | outcome_72h ~ age_years + gender + gcs_min_24h + vasopressor_24h + ventilation_24h + comorb_hypertension + comorb_diabetes + comorb_atrial_fibrillation + comorb_ihd + comorb_heart_failure + comorb_ckd + comorb_copd + comorb_malignancy + b_filab_01 + delta_filab_01 |

*Model E is the continuous baseline-and-change sensitivity model. Stable change and low-load stable phenotype are reference categories.*

**Supplementary Table S5. Complete development-cohort regression coefficients and intercepts.**

| **landmark** | **model** | **term** | **beta** | **standard_error** | **OR** | **CI_low** | **CI_high** | **z** | **p_value** |
| --- | --- | --- | --- | --- | --- | --- | --- | --- | --- |
| 24 h | Model_A | (Intercept) | -2.624 | 0.446 | 0.073 | 0.030 | 0.174 | -5.881 | <0.001 |
| 24 h | Model_A | age_years | 0.042 | 0.005 | 1.043 | 1.033 | 1.053 | 8.751 | <0.001 |
| 24 h | Model_A | genderM | 0.082 | 0.116 | 1.085 | 0.864 | 1.363 | 0.704 | 0.481 |
| 24 h | Model_A | gcs_min_24h | -0.166 | 0.021 | 0.847 | 0.812 | 0.883 | -7.777 | <0.001 |
| 24 h | Model_A | vasopressor_24h | -0.123 | 0.145 | 0.885 | 0.665 | 1.176 | -0.843 | 0.399 |
| 24 h | Model_A | ventilation_24h | -0.032 | 0.194 | 0.968 | 0.662 | 1.416 | -0.167 | 0.868 |
| 24 h | Model_A | comorb_hypertension | -0.269 | 0.147 | 0.764 | 0.573 | 1.019 | -1.833 | 0.067 |
| 24 h | Model_A | comorb_diabetes | -0.002 | 0.123 | 0.998 | 0.785 | 1.270 | -0.014 | 0.988 |
| 24 h | Model_A | comorb_atrial_fibrillation | -0.114 | 0.122 | 0.892 | 0.702 | 1.133 | -0.935 | 0.350 |
| 24 h | Model_A | comorb_ihd | 0.260 | 0.123 | 1.297 | 1.019 | 1.651 | 2.109 | 0.035 |
| 24 h | Model_A | comorb_heart_failure | 0.305 | 0.134 | 1.357 | 1.043 | 1.764 | 2.276 | 0.023 |
| 24 h | Model_A | comorb_ckd | 0.605 | 0.136 | 1.832 | 1.402 | 2.393 | 4.439 | <0.001 |
| 24 h | Model_A | comorb_copd | 0.211 | 0.165 | 1.234 | 0.894 | 1.705 | 1.278 | 0.201 |
| 24 h | Model_A | comorb_malignancy | 1.062 | 0.152 | 2.892 | 2.148 | 3.893 | 7.002 | <0.001 |
| 24 h | Model_B | (Intercept) | -3.775 | 0.482 | 0.023 | 0.009 | 0.059 | -7.824 | <0.001 |
| 24 h | Model_B | age_years | 0.045 | 0.005 | 1.046 | 1.036 | 1.056 | 9.083 | <0.001 |
| 24 h | Model_B | genderM | 0.113 | 0.119 | 1.120 | 0.887 | 1.413 | 0.952 | 0.341 |
| 24 h | Model_B | gcs_min_24h | -0.151 | 0.022 | 0.860 | 0.824 | 0.898 | -6.889 | <0.001 |
| 24 h | Model_B | vasopressor_24h | -0.250 | 0.148 | 0.779 | 0.582 | 1.042 | -1.682 | 0.093 |
| 24 h | Model_B | ventilation_24h | -0.076 | 0.198 | 0.926 | 0.629 | 1.365 | -0.387 | 0.699 |
| 24 h | Model_B | comorb_hypertension | -0.236 | 0.151 | 0.790 | 0.587 | 1.062 | -1.561 | 0.119 |
| 24 h | Model_B | comorb_diabetes | -0.147 | 0.126 | 0.863 | 0.674 | 1.105 | -1.167 | 0.243 |
| 24 h | Model_B | comorb_atrial_fibrillation | -0.104 | 0.124 | 0.901 | 0.706 | 1.150 | -0.836 | 0.403 |
| 24 h | Model_B | comorb_ihd | 0.215 | 0.125 | 1.240 | 0.970 | 1.585 | 1.721 | 0.085 |
| 24 h | Model_B | comorb_heart_failure | 0.196 | 0.137 | 1.217 | 0.931 | 1.590 | 1.436 | 0.151 |
| 24 h | Model_B | comorb_ckd | 0.174 | 0.147 | 1.190 | 0.893 | 1.588 | 1.186 | 0.236 |
| 24 h | Model_B | comorb_copd | 0.167 | 0.167 | 1.182 | 0.851 | 1.641 | 0.997 | 0.319 |
| 24 h | Model_B | comorb_malignancy | 0.864 | 0.155 | 2.373 | 1.752 | 3.213 | 5.582 | <0.001 |
| 24 h | Model_B | b_filab_01 | 0.277 | 0.034 | 1.319 | 1.235 | 1.409 | 8.238 | <0.001 |
| 72 h | Model_A | (Intercept) | -3.146 | 0.472 | 0.043 | 0.017 | 0.109 | -6.665 | <0.001 |
| 72 h | Model_A | age_years | 0.040 | 0.005 | 1.041 | 1.030 | 1.051 | 7.947 | <0.001 |
| 72 h | Model_A | genderM | 0.151 | 0.122 | 1.163 | 0.916 | 1.477 | 1.242 | 0.214 |
| 72 h | Model_A | gcs_min_24h | -0.124 | 0.023 | 0.883 | 0.845 | 0.924 | -5.461 | <0.001 |
| 72 h | Model_A | vasopressor_24h | -0.005 | 0.151 | 0.995 | 0.740 | 1.339 | -0.030 | 0.976 |
| 72 h | Model_A | ventilation_24h | 0.150 | 0.207 | 1.162 | 0.775 | 1.742 | 0.726 | 0.468 |
| 72 h | Model_A | comorb_hypertension | -0.264 | 0.156 | 0.768 | 0.566 | 1.043 | -1.688 | 0.091 |
| 72 h | Model_A | comorb_diabetes | 0.030 | 0.129 | 1.030 | 0.801 | 1.325 | 0.230 | 0.818 |
| 72 h | Model_A | comorb_atrial_fibrillation | -0.151 | 0.128 | 0.860 | 0.668 | 1.105 | -1.180 | 0.238 |
| 72 h | Model_A | comorb_ihd | 0.245 | 0.129 | 1.278 | 0.992 | 1.646 | 1.898 | 0.058 |
| 72 h | Model_A | comorb_heart_failure | 0.253 | 0.141 | 1.287 | 0.977 | 1.697 | 1.792 | 0.073 |
| 72 h | Model_A | comorb_ckd | 0.711 | 0.142 | 2.037 | 1.543 | 2.689 | 5.022 | <0.001 |
| 72 h | Model_A | comorb_copd | 0.231 | 0.173 | 1.260 | 0.897 | 1.770 | 1.332 | 0.183 |
| 72 h | Model_A | comorb_malignancy | 1.034 | 0.158 | 2.811 | 2.061 | 3.835 | 6.526 | <0.001 |
| 72 h | Model_B | (Intercept) | -4.263 | 0.509 | 0.014 | 0.005 | 0.038 | -8.368 | <0.001 |
| 72 h | Model_B | age_years | 0.043 | 0.005 | 1.044 | 1.033 | 1.055 | 8.309 | <0.001 |
| 72 h | Model_B | genderM | 0.193 | 0.124 | 1.213 | 0.951 | 1.547 | 1.558 | 0.119 |
| 72 h | Model_B | gcs_min_24h | -0.111 | 0.023 | 0.895 | 0.855 | 0.936 | -4.803 | <0.001 |
| 72 h | Model_B | vasopressor_24h | -0.132 | 0.154 | 0.876 | 0.647 | 1.186 | -0.857 | 0.391 |
| 72 h | Model_B | ventilation_24h | 0.097 | 0.210 | 1.102 | 0.730 | 1.663 | 0.462 | 0.644 |
| 72 h | Model_B | comorb_hypertension | -0.246 | 0.160 | 0.782 | 0.571 | 1.070 | -1.536 | 0.125 |
| 72 h | Model_B | comorb_diabetes | -0.119 | 0.132 | 0.888 | 0.685 | 1.150 | -0.902 | 0.367 |
| 72 h | Model_B | comorb_atrial_fibrillation | -0.143 | 0.131 | 0.867 | 0.671 | 1.120 | -1.096 | 0.273 |
| 72 h | Model_B | comorb_ihd | 0.205 | 0.131 | 1.228 | 0.949 | 1.588 | 1.565 | 0.118 |
| 72 h | Model_B | comorb_heart_failure | 0.162 | 0.144 | 1.175 | 0.887 | 1.557 | 1.125 | 0.260 |
| 72 h | Model_B | comorb_ckd | 0.291 | 0.153 | 1.337 | 0.992 | 1.803 | 1.904 | 0.057 |
| 72 h | Model_B | comorb_copd | 0.180 | 0.176 | 1.197 | 0.848 | 1.689 | 1.022 | 0.307 |
| 72 h | Model_B | comorb_malignancy | 0.842 | 0.162 | 2.322 | 1.692 | 3.187 | 5.212 | <0.001 |
| 72 h | Model_B | b_filab_01 | 0.271 | 0.035 | 1.311 | 1.223 | 1.405 | 7.685 | <0.001 |
| 72 h | Model_C | (Intercept) | -4.672 | 0.524 | 0.009 | 0.003 | 0.026 | -8.918 | <0.001 |
| 72 h | Model_C | age_years | 0.044 | 0.005 | 1.045 | 1.034 | 1.056 | 8.374 | <0.001 |
| 72 h | Model_C | genderM | 0.217 | 0.125 | 1.242 | 0.972 | 1.588 | 1.733 | 0.083 |
| 72 h | Model_C | gcs_min_24h | -0.101 | 0.023 | 0.904 | 0.863 | 0.946 | -4.308 | <0.001 |
| 72 h | Model_C | vasopressor_24h | -0.166 | 0.156 | 0.847 | 0.624 | 1.150 | -1.063 | 0.288 |
| 72 h | Model_C | ventilation_24h | 0.074 | 0.213 | 1.077 | 0.710 | 1.634 | 0.348 | 0.728 |
| 72 h | Model_C | comorb_hypertension | -0.259 | 0.163 | 0.772 | 0.561 | 1.061 | -1.595 | 0.111 |
| 72 h | Model_C | comorb_diabetes | -0.121 | 0.133 | 0.886 | 0.683 | 1.151 | -0.906 | 0.365 |
| 72 h | Model_C | comorb_atrial_fibrillation | -0.148 | 0.132 | 0.863 | 0.666 | 1.118 | -1.117 | 0.264 |
| 72 h | Model_C | comorb_ihd | 0.226 | 0.132 | 1.253 | 0.967 | 1.625 | 1.705 | 0.088 |
| 72 h | Model_C | comorb_heart_failure | 0.116 | 0.145 | 1.123 | 0.845 | 1.492 | 0.800 | 0.424 |
| 72 h | Model_C | comorb_ckd | 0.170 | 0.156 | 1.185 | 0.873 | 1.608 | 1.089 | 0.276 |
| 72 h | Model_C | comorb_copd | 0.126 | 0.178 | 1.135 | 0.801 | 1.607 | 0.711 | 0.477 |
| 72 h | Model_C | comorb_malignancy | 0.825 | 0.164 | 2.281 | 1.655 | 3.144 | 5.035 | <0.001 |
| 72 h | Model_C | b_filab_01 | 0.364 | 0.040 | 1.439 | 1.330 | 1.558 | 9.053 | <0.001 |
| 72 h | Model_C | change_dirImproved | -0.843 | 0.196 | 0.430 | 0.293 | 0.631 | -4.312 | <0.001 |
| 72 h | Model_C | change_dirWorsened | 0.431 | 0.172 | 1.539 | 1.099 | 2.156 | 2.506 | 0.012 |
| 72 h | Model_D | (Intercept) | -3.925 | 0.499 | 0.020 | 0.007 | 0.052 | -7.873 | <0.001 |
| 72 h | Model_D | age_years | 0.039 | 0.005 | 1.040 | 1.030 | 1.051 | 7.749 | <0.001 |
| 72 h | Model_D | genderM | 0.176 | 0.123 | 1.193 | 0.937 | 1.518 | 1.432 | 0.152 |
| 72 h | Model_D | gcs_min_24h | -0.108 | 0.023 | 0.897 | 0.858 | 0.939 | -4.698 | <0.001 |
| 72 h | Model_D | vasopressor_24h | -0.052 | 0.152 | 0.950 | 0.705 | 1.280 | -0.339 | 0.734 |
| 72 h | Model_D | ventilation_24h | 0.092 | 0.209 | 1.097 | 0.728 | 1.654 | 0.442 | 0.659 |
| 72 h | Model_D | comorb_hypertension | -0.291 | 0.159 | 0.747 | 0.547 | 1.021 | -1.828 | 0.068 |
| 72 h | Model_D | comorb_diabetes | -0.010 | 0.130 | 0.990 | 0.768 | 1.277 | -0.075 | 0.941 |
| 72 h | Model_D | comorb_atrial_fibrillation | -0.183 | 0.130 | 0.833 | 0.646 | 1.075 | -1.405 | 0.160 |
| 72 h | Model_D | comorb_ihd | 0.239 | 0.131 | 1.270 | 0.983 | 1.641 | 1.829 | 0.067 |
| 72 h | Model_D | comorb_heart_failure | 0.188 | 0.142 | 1.207 | 0.913 | 1.595 | 1.323 | 0.186 |
| 72 h | Model_D | comorb_ckd | 0.499 | 0.146 | 1.648 | 1.237 | 2.195 | 3.412 | <0.001 |
| 72 h | Model_D | comorb_copd | 0.200 | 0.175 | 1.222 | 0.868 | 1.720 | 1.147 | 0.252 |
| 72 h | Model_D | comorb_malignancy | 0.914 | 0.161 | 2.493 | 1.819 | 3.419 | 5.674 | <0.001 |
| 72 h | Model_D | dynamic_phenotypeLow-load worsened | 0.895 | 0.277 | 2.447 | 1.423 | 4.209 | 3.235 | 0.001 |
| 72 h | Model_D | dynamic_phenotypeHigh-load improved | 0.736 | 0.236 | 2.088 | 1.314 | 3.318 | 3.115 | 0.002 |
| 72 h | Model_D | dynamic_phenotypeHigh-load persistent | 1.143 | 0.188 | 3.137 | 2.170 | 4.535 | 6.080 | <0.001 |
| 72 h | Model_E | (Intercept) | -4.832 | 0.528 | 0.008 | 0.003 | 0.022 | -9.159 | <0.001 |
| 72 h | Model_E | age_years | 0.044 | 0.005 | 1.045 | 1.034 | 1.056 | 8.316 | <0.001 |
| 72 h | Model_E | genderM | 0.210 | 0.126 | 1.234 | 0.965 | 1.578 | 1.673 | 0.094 |
| 72 h | Model_E | gcs_min_24h | -0.095 | 0.024 | 0.909 | 0.868 | 0.952 | -4.049 | <0.001 |
| 72 h | Model_E | vasopressor_24h | -0.177 | 0.157 | 0.838 | 0.616 | 1.139 | -1.131 | 0.258 |
| 72 h | Model_E | ventilation_24h | 0.049 | 0.213 | 1.050 | 0.692 | 1.595 | 0.230 | 0.818 |
| 72 h | Model_E | comorb_hypertension | -0.257 | 0.163 | 0.774 | 0.561 | 1.066 | -1.571 | 0.116 |
| 72 h | Model_E | comorb_diabetes | -0.150 | 0.134 | 0.861 | 0.662 | 1.118 | -1.122 | 0.262 |
| 72 h | Model_E | comorb_atrial_fibrillation | -0.169 | 0.132 | 0.845 | 0.652 | 1.095 | -1.274 | 0.203 |
| 72 h | Model_E | comorb_ihd | 0.213 | 0.133 | 1.237 | 0.953 | 1.606 | 1.600 | 0.110 |
| 72 h | Model_E | comorb_heart_failure | 0.099 | 0.146 | 1.104 | 0.830 | 1.469 | 0.681 | 0.496 |
| 72 h | Model_E | comorb_ckd | 0.156 | 0.156 | 1.169 | 0.861 | 1.586 | 1.001 | 0.317 |
| 72 h | Model_E | comorb_copd | 0.139 | 0.178 | 1.149 | 0.811 | 1.629 | 0.782 | 0.434 |
| 72 h | Model_E | comorb_malignancy | 0.786 | 0.164 | 2.194 | 1.590 | 3.029 | 4.778 | <0.001 |
| 72 h | Model_E | b_filab_01 | 0.393 | 0.041 | 1.481 | 1.366 | 1.605 | 9.542 | <0.001 |
| 72 h | Model_E | delta_filab_01 | 0.283 | 0.046 | 1.327 | 1.213 | 1.451 | 6.204 | <0.001 |

*All coefficients, standard errors, odds ratios, confidence intervals, z statistics, and P values are shown; intercept odds ratios are retained for computational completeness.*

**Supplementary Table S6. Performance of all models at both landmarks.**

| **landmark** | **dataset** | **model** | **n** | **events** | **event_rate** | **brier** | **mean_predicted_risk** | **auc** | **auc_low** | **auc_high** | **calibration_intercept** | **calibration_slope** |
| --- | --- | --- | --- | --- | --- | --- | --- | --- | --- | --- | --- | --- |
| 24 h | development | Model_A | 1,890 | 559 | 0.296 | 0.171 | 0.296 | 0.767 | 0.745 | 0.790 | 0.000 | 1.000 |
| 24 h | development | Model_B | 1,890 | 559 | 0.296 | 0.165 | 0.296 | 0.788 | 0.766 | 0.809 | 0.000 | 1.000 |
| 24 h | temporal_validation | Model_A | 1,296 | 339 | 0.262 | 0.162 | 0.247 | 0.774 | 0.747 | 0.801 | 0.098 | 0.941 |
| 24 h | temporal_validation | Model_B | 1,296 | 339 | 0.262 | 0.152 | 0.248 | 0.808 | 0.784 | 0.833 | 0.095 | 0.943 |
| 72 h | development | Model_A | 1,754 | 473 | 0.270 | 0.167 | 0.270 | 0.755 | 0.731 | 0.779 | 0.000 | 1.000 |
| 72 h | development | Model_B | 1,754 | 473 | 0.270 | 0.161 | 0.270 | 0.776 | 0.753 | 0.799 | 0.000 | 1.000 |
| 72 h | development | Model_C | 1,754 | 473 | 0.270 | 0.158 | 0.270 | 0.787 | 0.765 | 0.810 | 0.000 | 1.000 |
| 72 h | development | Model_D | 1,754 | 473 | 0.270 | 0.163 | 0.270 | 0.768 | 0.745 | 0.792 | 0.000 | 1.000 |
| 72 h | development | Model_E | 1,754 | 473 | 0.270 | 0.157 | 0.270 | 0.793 | 0.771 | 0.815 | 0.000 | 1.000 |
| 72 h | temporal_validation | Model_A | 1,164 | 287 | 0.247 | 0.163 | 0.227 | 0.752 | 0.722 | 0.782 | 0.129 | 0.916 |
| 72 h | temporal_validation | Model_B | 1,164 | 287 | 0.247 | 0.152 | 0.229 | 0.791 | 0.763 | 0.819 | 0.120 | 0.938 |
| 72 h | temporal_validation | Model_C | 1,164 | 287 | 0.247 | 0.150 | 0.231 | 0.797 | 0.769 | 0.825 | 0.114 | 0.910 |
| 72 h | temporal_validation | Model_D | 1,164 | 287 | 0.247 | 0.159 | 0.223 | 0.767 | 0.738 | 0.795 | 0.161 | 0.902 |
| 72 h | temporal_validation | Model_E | 1,164 | 287 | 0.247 | 0.150 | 0.232 | 0.798 | 0.770 | 0.825 | 0.104 | 0.906 |

*Models were fitted in development and applied without refitting to temporal validation.*

**Supplementary Table S7. Paired bootstrap comparisons in temporal validation.**

| **landmark** | **dataset** | **reference_model** | **compared_model** | **metric** | **estimate** | **CI_low** | **CI_high** | **bootstrap_p** | **successful_replicates** | **bootstrap_reps** |
| --- | --- | --- | --- | --- | --- | --- | --- | --- | --- | --- |
| 24 h | temporal_validation | Model_A | Model_B | AUC difference | 0.034 | 0.022 | 0.047 | <0.001 | 2,000 | 2,000 |
| 24 h | temporal_validation | Model_A | Model_B | Brier difference | -0.011 | -0.015 | -0.006 | <0.001 | 2,000 | 2,000 |
| 72 h | temporal_validation | Model_A | Model_B | AUC difference | 0.039 | 0.025 | 0.054 | <0.001 | 2,000 | 2,000 |
| 72 h | temporal_validation | Model_A | Model_B | Brier difference | -0.011 | -0.015 | -0.007 | <0.001 | 2,000 | 2,000 |
| 72 h | temporal_validation | Model_B | Model_C | AUC difference | 0.006 | -0.003 | 0.014 | 0.184 | 2,000 | 2,000 |
| 72 h | temporal_validation | Model_B | Model_C | Brier difference | -0.002 | -0.005 | 0.001 | 0.247 | 2,000 | 2,000 |
| 72 h | temporal_validation | Model_B | Model_D | AUC difference | -0.024 | -0.037 | -0.012 | <0.001 | 2,000 | 2,000 |
| 72 h | temporal_validation | Model_B | Model_D | Brier difference | 0.007 | 0.003 | 0.011 | <0.001 | 2,000 | 2,000 |
| 72 h | temporal_validation | Model_B | Model_E | AUC difference | 0.006 | -0.003 | 0.017 | 0.192 | 2,000 | 2,000 |
| 72 h | temporal_validation | Model_B | Model_E | Brier difference | -0.002 | -0.005 | 0.002 | 0.333 | 2,000 | 2,000 |

*Positive AUC differences and negative Brier differences favor the compared model. All 2,000 replicates completed successfully.*

**Supplementary Table S8A. Conventional variance inflation factors.**

| **landmark** | **model** | **design_column** | **VIF** |
| --- | --- | --- | --- |
| 24 h | Model_A | age_years | 1.360 |
| 24 h | Model_A | genderM | 1.068 |
| 24 h | Model_A | gcs_min_24h | 3.254 |
| 24 h | Model_A | vasopressor_24h | 1.379 |
| 24 h | Model_A | ventilation_24h | 3.249 |
| 24 h | Model_A | comorb_hypertension | 1.181 |
| 24 h | Model_A | comorb_diabetes | 1.119 |
| 24 h | Model_A | comorb_atrial_fibrillation | 1.219 |
| 24 h | Model_A | comorb_ihd | 1.203 |
| 24 h | Model_A | comorb_heart_failure | 1.284 |
| 24 h | Model_A | comorb_ckd | 1.212 |
| 24 h | Model_A | comorb_copd | 1.029 |
| 24 h | Model_A | comorb_malignancy | 1.008 |
| 24 h | Model_B | age_years | 1.360 |
| 24 h | Model_B | genderM | 1.072 |
| 24 h | Model_B | gcs_min_24h | 3.311 |
| 24 h | Model_B | vasopressor_24h | 1.396 |
| 24 h | Model_B | ventilation_24h | 3.251 |
| 24 h | Model_B | comorb_hypertension | 1.182 |
| 24 h | Model_B | comorb_diabetes | 1.142 |
| 24 h | Model_B | comorb_atrial_fibrillation | 1.220 |
| 24 h | Model_B | comorb_ihd | 1.208 |
| 24 h | Model_B | comorb_heart_failure | 1.299 |
| 24 h | Model_B | comorb_ckd | 1.397 |
| 24 h | Model_B | comorb_copd | 1.032 |
| 24 h | Model_B | comorb_malignancy | 1.043 |
| 24 h | Model_B | b_filab_01 | 1.490 |
| 72 h | Model_A | age_years | 1.356 |
| 72 h | Model_A | genderM | 1.064 |
| 72 h | Model_A | gcs_min_24h | 3.360 |
| 72 h | Model_A | vasopressor_24h | 1.388 |
| 72 h | Model_A | ventilation_24h | 3.324 |
| 72 h | Model_A | comorb_hypertension | 1.188 |
| 72 h | Model_A | comorb_diabetes | 1.121 |
| 72 h | Model_A | comorb_atrial_fibrillation | 1.216 |
| 72 h | Model_A | comorb_ihd | 1.206 |
| 72 h | Model_A | comorb_heart_failure | 1.293 |
| 72 h | Model_A | comorb_ckd | 1.217 |
| 72 h | Model_A | comorb_copd | 1.029 |
| 72 h | Model_A | comorb_malignancy | 1.009 |
| 72 h | Model_B | age_years | 1.356 |
| 72 h | Model_B | genderM | 1.069 |
| 72 h | Model_B | gcs_min_24h | 3.401 |
| 72 h | Model_B | vasopressor_24h | 1.406 |
| 72 h | Model_B | ventilation_24h | 3.328 |
| 72 h | Model_B | comorb_hypertension | 1.189 |
| 72 h | Model_B | comorb_diabetes | 1.146 |
| 72 h | Model_B | comorb_atrial_fibrillation | 1.216 |
| 72 h | Model_B | comorb_ihd | 1.211 |
| 72 h | Model_B | comorb_heart_failure | 1.305 |
| 72 h | Model_B | comorb_ckd | 1.412 |
| 72 h | Model_B | comorb_copd | 1.031 |
| 72 h | Model_B | comorb_malignancy | 1.044 |
| 72 h | Model_B | b_filab_01 | 1.486 |
| 72 h | Model_C | age_years | 1.361 |
| 72 h | Model_C | genderM | 1.073 |
| 72 h | Model_C | gcs_min_24h | 3.438 |
| 72 h | Model_C | vasopressor_24h | 1.408 |
| 72 h | Model_C | ventilation_24h | 3.337 |
| 72 h | Model_C | comorb_hypertension | 1.190 |
| 72 h | Model_C | comorb_diabetes | 1.150 |
| 72 h | Model_C | comorb_atrial_fibrillation | 1.218 |
| 72 h | Model_C | comorb_ihd | 1.211 |
| 72 h | Model_C | comorb_heart_failure | 1.310 |
| 72 h | Model_C | comorb_ckd | 1.437 |
| 72 h | Model_C | comorb_copd | 1.034 |
| 72 h | Model_C | comorb_malignancy | 1.046 |
| 72 h | Model_C | b_filab_01 | 1.816 |
| 72 h | Model_C | change_dirImproved | 1.196 |
| 72 h | Model_C | change_dirWorsened | 1.110 |
| 72 h | Model_D | age_years | 1.362 |
| 72 h | Model_D | genderM | 1.069 |
| 72 h | Model_D | gcs_min_24h | 3.414 |
| 72 h | Model_D | vasopressor_24h | 1.395 |
| 72 h | Model_D | ventilation_24h | 3.333 |
| 72 h | Model_D | comorb_hypertension | 1.189 |
| 72 h | Model_D | comorb_diabetes | 1.134 |
| 72 h | Model_D | comorb_atrial_fibrillation | 1.217 |
| 72 h | Model_D | comorb_ihd | 1.211 |
| 72 h | Model_D | comorb_heart_failure | 1.302 |
| 72 h | Model_D | comorb_ckd | 1.312 |
| 72 h | Model_D | comorb_copd | 1.030 |
| 72 h | Model_D | comorb_malignancy | 1.036 |
| 72 h | Model_D | dynamic_phenotypeLow-load worsened | 1.205 |
| 72 h | Model_D | dynamic_phenotypeHigh-load improved | 1.392 |
| 72 h | Model_D | dynamic_phenotypeHigh-load persistent | 1.765 |
| 72 h | Model_E | age_years | 1.357 |
| 72 h | Model_E | genderM | 1.070 |
| 72 h | Model_E | gcs_min_24h | 3.453 |
| 72 h | Model_E | vasopressor_24h | 1.408 |
| 72 h | Model_E | ventilation_24h | 3.338 |
| 72 h | Model_E | comorb_hypertension | 1.189 |
| 72 h | Model_E | comorb_diabetes | 1.148 |
| 72 h | Model_E | comorb_atrial_fibrillation | 1.216 |
| 72 h | Model_E | comorb_ihd | 1.211 |
| 72 h | Model_E | comorb_heart_failure | 1.312 |
| 72 h | Model_E | comorb_ckd | 1.439 |
| 72 h | Model_E | comorb_copd | 1.033 |
| 72 h | Model_E | comorb_malignancy | 1.050 |
| 72 h | Model_E | b_filab_01 | 1.885 |
| 72 h | Model_E | delta_filab_01 | 1.307 |

*The maximum VIF was 3.45; the baseline FI-Lab VIF in the 72-hour Model B was 1.48.*

**Supplementary Table S8B. Generalized variance inflation factors for factor terms.**

| **landmark** | **model** | **term** | **degrees_freedom** | **GVIF** | **GVIF_adjusted** |
| --- | --- | --- | --- | --- | --- |
| 24 h | Model_A | age_years | 1 | 1.360 | 1.166 |
| 24 h | Model_A | gender | 1 | 1.068 | 1.033 |
| 24 h | Model_A | gcs_min_24h | 1 | 3.254 | 1.804 |
| 24 h | Model_A | vasopressor_24h | 1 | 1.379 | 1.174 |
| 24 h | Model_A | ventilation_24h | 1 | 3.249 | 1.803 |
| 24 h | Model_A | comorb_hypertension | 1 | 1.181 | 1.087 |
| 24 h | Model_A | comorb_diabetes | 1 | 1.119 | 1.058 |
| 24 h | Model_A | comorb_atrial_fibrillation | 1 | 1.219 | 1.104 |
| 24 h | Model_A | comorb_ihd | 1 | 1.203 | 1.097 |
| 24 h | Model_A | comorb_heart_failure | 1 | 1.284 | 1.133 |
| 24 h | Model_A | comorb_ckd | 1 | 1.212 | 1.101 |
| 24 h | Model_A | comorb_copd | 1 | 1.029 | 1.015 |
| 24 h | Model_A | comorb_malignancy | 1 | 1.008 | 1.004 |
| 24 h | Model_B | age_years | 1 | 1.360 | 1.166 |
| 24 h | Model_B | gender | 1 | 1.072 | 1.035 |
| 24 h | Model_B | gcs_min_24h | 1 | 3.311 | 1.820 |
| 24 h | Model_B | vasopressor_24h | 1 | 1.396 | 1.181 |
| 24 h | Model_B | ventilation_24h | 1 | 3.251 | 1.803 |
| 24 h | Model_B | comorb_hypertension | 1 | 1.182 | 1.087 |
| 24 h | Model_B | comorb_diabetes | 1 | 1.142 | 1.069 |
| 24 h | Model_B | comorb_atrial_fibrillation | 1 | 1.220 | 1.104 |
| 24 h | Model_B | comorb_ihd | 1 | 1.208 | 1.099 |
| 24 h | Model_B | comorb_heart_failure | 1 | 1.299 | 1.140 |
| 24 h | Model_B | comorb_ckd | 1 | 1.397 | 1.182 |
| 24 h | Model_B | comorb_copd | 1 | 1.032 | 1.016 |
| 24 h | Model_B | comorb_malignancy | 1 | 1.043 | 1.022 |
| 24 h | Model_B | b_filab_01 | 1 | 1.490 | 1.221 |
| 72 h | Model_A | age_years | 1 | 1.356 | 1.164 |
| 72 h | Model_A | gender | 1 | 1.064 | 1.031 |
| 72 h | Model_A | gcs_min_24h | 1 | 3.360 | 1.833 |
| 72 h | Model_A | vasopressor_24h | 1 | 1.388 | 1.178 |
| 72 h | Model_A | ventilation_24h | 1 | 3.324 | 1.823 |
| 72 h | Model_A | comorb_hypertension | 1 | 1.188 | 1.090 |
| 72 h | Model_A | comorb_diabetes | 1 | 1.121 | 1.059 |
| 72 h | Model_A | comorb_atrial_fibrillation | 1 | 1.216 | 1.103 |
| 72 h | Model_A | comorb_ihd | 1 | 1.206 | 1.098 |
| 72 h | Model_A | comorb_heart_failure | 1 | 1.293 | 1.137 |
| 72 h | Model_A | comorb_ckd | 1 | 1.217 | 1.103 |
| 72 h | Model_A | comorb_copd | 1 | 1.029 | 1.014 |
| 72 h | Model_A | comorb_malignancy | 1 | 1.009 | 1.004 |
| 72 h | Model_B | age_years | 1 | 1.356 | 1.165 |
| 72 h | Model_B | gender | 1 | 1.069 | 1.034 |
| 72 h | Model_B | gcs_min_24h | 1 | 3.401 | 1.844 |
| 72 h | Model_B | vasopressor_24h | 1 | 1.406 | 1.186 |
| 72 h | Model_B | ventilation_24h | 1 | 3.328 | 1.824 |
| 72 h | Model_B | comorb_hypertension | 1 | 1.189 | 1.090 |
| 72 h | Model_B | comorb_diabetes | 1 | 1.146 | 1.071 |
| 72 h | Model_B | comorb_atrial_fibrillation | 1 | 1.216 | 1.103 |
| 72 h | Model_B | comorb_ihd | 1 | 1.211 | 1.100 |
| 72 h | Model_B | comorb_heart_failure | 1 | 1.305 | 1.142 |
| 72 h | Model_B | comorb_ckd | 1 | 1.412 | 1.188 |
| 72 h | Model_B | comorb_copd | 1 | 1.031 | 1.016 |
| 72 h | Model_B | comorb_malignancy | 1 | 1.044 | 1.022 |
| 72 h | Model_B | b_filab_01 | 1 | 1.486 | 1.219 |
| 72 h | Model_C | age_years | 1 | 1.361 | 1.167 |
| 72 h | Model_C | gender | 1 | 1.073 | 1.036 |
| 72 h | Model_C | gcs_min_24h | 1 | 3.438 | 1.854 |
| 72 h | Model_C | vasopressor_24h | 1 | 1.408 | 1.187 |
| 72 h | Model_C | ventilation_24h | 1 | 3.337 | 1.827 |
| 72 h | Model_C | comorb_hypertension | 1 | 1.190 | 1.091 |
| 72 h | Model_C | comorb_diabetes | 1 | 1.150 | 1.072 |
| 72 h | Model_C | comorb_atrial_fibrillation | 1 | 1.218 | 1.104 |
| 72 h | Model_C | comorb_ihd | 1 | 1.211 | 1.100 |
| 72 h | Model_C | comorb_heart_failure | 1 | 1.310 | 1.145 |
| 72 h | Model_C | comorb_ckd | 1 | 1.437 | 1.199 |
| 72 h | Model_C | comorb_copd | 1 | 1.034 | 1.017 |
| 72 h | Model_C | comorb_malignancy | 1 | 1.046 | 1.023 |
| 72 h | Model_C | b_filab_01 | 1 | 1.816 | 1.347 |
| 72 h | Model_C | change_dir | 2 | 1.285 | 1.065 |
| 72 h | Model_D | age_years | 1 | 1.362 | 1.167 |
| 72 h | Model_D | gender | 1 | 1.069 | 1.034 |
| 72 h | Model_D | gcs_min_24h | 1 | 3.414 | 1.848 |
| 72 h | Model_D | vasopressor_24h | 1 | 1.395 | 1.181 |
| 72 h | Model_D | ventilation_24h | 1 | 3.333 | 1.826 |
| 72 h | Model_D | comorb_hypertension | 1 | 1.189 | 1.090 |
| 72 h | Model_D | comorb_diabetes | 1 | 1.134 | 1.065 |
| 72 h | Model_D | comorb_atrial_fibrillation | 1 | 1.217 | 1.103 |
| 72 h | Model_D | comorb_ihd | 1 | 1.211 | 1.100 |
| 72 h | Model_D | comorb_heart_failure | 1 | 1.302 | 1.141 |
| 72 h | Model_D | comorb_ckd | 1 | 1.312 | 1.146 |
| 72 h | Model_D | comorb_copd | 1 | 1.030 | 1.015 |
| 72 h | Model_D | comorb_malignancy | 1 | 1.036 | 1.018 |
| 72 h | Model_D | dynamic_phenotype | 3 | 1.354 | 1.052 |
| 72 h | Model_E | age_years | 1 | 1.357 | 1.165 |
| 72 h | Model_E | gender | 1 | 1.070 | 1.034 |
| 72 h | Model_E | gcs_min_24h | 1 | 3.453 | 1.858 |
| 72 h | Model_E | vasopressor_24h | 1 | 1.408 | 1.187 |
| 72 h | Model_E | ventilation_24h | 1 | 3.338 | 1.827 |
| 72 h | Model_E | comorb_hypertension | 1 | 1.189 | 1.090 |
| 72 h | Model_E | comorb_diabetes | 1 | 1.148 | 1.071 |
| 72 h | Model_E | comorb_atrial_fibrillation | 1 | 1.216 | 1.103 |
| 72 h | Model_E | comorb_ihd | 1 | 1.211 | 1.100 |
| 72 h | Model_E | comorb_heart_failure | 1 | 1.312 | 1.145 |
| 72 h | Model_E | comorb_ckd | 1 | 1.439 | 1.199 |
| 72 h | Model_E | comorb_copd | 1 | 1.033 | 1.016 |
| 72 h | Model_E | comorb_malignancy | 1 | 1.050 | 1.025 |
| 72 h | Model_E | b_filab_01 | 1 | 1.885 | 1.373 |
| 72 h | Model_E | delta_filab_01 | 1 | 1.307 | 1.143 |

**Supplementary Table S9. Comparison of dynamically evaluable and non-evaluable 72-hour survivors.**

| **variable** | **type** | **evaluable_n** | **evaluable_summary** | **nonevaluable_n** | **nonevaluable_summary** | **standardized_difference** |
| --- | --- | --- | --- | --- | --- | --- |
| age_years | continuous | 2,930 | 68.991 (15.246) | 168 | 70.399 (16.040) | -0.090 |
| male_indicator | binary | 2,930 | 1499/2930 (51.2%) | 168 | 73/168 (43.5%) | 0.155 |
| gcs_min_24h | continuous | 2,918 | 10.170 (4.582) | 168 | 12.923 (3.187) | -0.698 |
| vasopressor_24h | binary | 2,930 | 669/2930 (22.8%) | 168 | 6/168 (3.6%) | 0.594 |
| ventilation_24h | binary | 2,930 | 1067/2930 (36.4%) | 168 | 9/168 (5.4%) | 0.827 |
| comorb_hypertension | binary | 2,930 | 2224/2930 (75.9%) | 168 | 113/168 (67.3%) | 0.193 |
| comorb_diabetes | binary | 2,930 | 970/2930 (33.1%) | 168 | 37/168 (22.0%) | 0.250 |
| comorb_atrial_fibrillation | binary | 2,930 | 1105/2930 (37.7%) | 168 | 51/168 (30.4%) | 0.156 |
| comorb_ihd | binary | 2,930 | 960/2930 (32.8%) | 168 | 35/168 (20.8%) | 0.272 |
| comorb_heart_failure | binary | 2,930 | 761/2930 (26.0%) | 168 | 20/168 (11.9%) | 0.365 |
| comorb_ckd | binary | 2,930 | 610/2930 (20.8%) | 168 | 24/168 (14.3%) | 0.172 |
| comorb_copd | binary | 2,930 | 314/2930 (10.7%) | 168 | 18/168 (10.7%) | 0.000 |
| comorb_malignancy | binary | 2,930 | 377/2930 (12.9%) | 168 | 21/168 (12.5%) | 0.011 |
| b_filab | continuous | 2,930 | 0.335 (0.209) | 168 | 0.196 (0.185) | 0.704 |
| icu_discharge_before_72h | binary | 2,930 | 1326/2930 (45.3%) | 168 | 152/168 (90.5%) | -1.107 |
| hospital_discharge_before_72h | binary | 2,930 | 277/2930 (9.5%) | 168 | 105/168 (62.5%) | -1.326 |
| outcome_72h | binary | 2,930 | 765/2930 (26.1%) | 168 | 38/168 (22.6%) | 0.081 |

*Standardized differences are reported to characterize selection related to repeat testing; they are not causal estimates.*

**Supplementary Table S10. Alternative phenotype-cutoff sensitivity analyses.**

| **high_load_cutoff** | **change_cutoff** | **development_n** | **development_events** | **validation_n** | **validation_events** | **model_converged** | **predictions_valid** | **validation_auc** | **validation_brier** |
| --- | --- | --- | --- | --- | --- | --- | --- | --- | --- |
| 0.200 | 0.100 | 1,754 | 473 | 1,164 | 287 | Yes | Yes | 0.749 | 0.162 |
| 0.200 | 0.125 | 1,754 | 473 | 1,164 | 287 | Yes | Yes | 0.749 | 0.162 |
| 0.200 | 0.150 | 1,754 | 473 | 1,164 | 287 | Yes | Yes | 0.749 | 0.162 |
| 0.300 | 0.100 | 1,754 | 473 | 1,164 | 287 | Yes | Yes | 0.767 | 0.158 |
| 0.300 | 0.125 | 1,754 | 473 | 1,164 | 287 | Yes | Yes | 0.767 | 0.159 |
| 0.300 | 0.150 | 1,754 | 473 | 1,164 | 287 | Yes | Yes | 0.766 | 0.159 |
| 0.400 | 0.100 | 1,754 | 473 | 1,164 | 287 | Yes | Yes | 0.775 | 0.157 |
| 0.400 | 0.125 | 1,754 | 473 | 1,164 | 287 | Yes | Yes | 0.776 | 0.156 |
| 0.400 | 0.150 | 1,754 | 473 | 1,164 | 287 | Yes | Yes | 0.776 | 0.156 |

*All combinations of high-load cutoffs 0.20/0.30/0.40 and change cutoffs 0.10/0.125/0.15 converged.*

**Supplementary Table S11A. Matched-domain model-performance sensitivity analysis.**

| **analysis** | **development_n** | **development_events** | **validation_n** | **validation_events** | **model_converged** | **predictions_valid** | **validation_auc** | **validation_brier** |
| --- | --- | --- | --- | --- | --- | --- | --- | --- |
| Same domains at baseline and follow-up; >=6 matched domains | 1,754 | 473 | 1,164 | 287 | Yes | Yes | 0.767 | 0.159 |

*Change was recalculated using at least six laboratory domains available at both time points. Agreement metrics and phenotype cross-classification are reported in Supplementary Tables S11B and S11C.*

**Supplementary Table S11B. Agreement between primary and matched-domain FI-Lab change and phenotype classifications.**

| **Metric** | **Value** | **Unit** |
| --- | --- | --- |
| Dynamically evaluable, primary rule | 2,930 | n |
| Dynamically evaluable, >=6 matched domains | 2,930 | n |
| Identical baseline/follow-up availability pattern | 97.7% | proportion |
| Correlation: primary vs matched delta | 0.995 | Pearson r |
| Jointly classifiable in primary and matched-domain analyses | 2,930 | n |
| Phenotype agreement among jointly classifiable patients | 99.5% | proportion |
| Expected agreement by chance | 36.0% | proportion |
| Cohen's kappa for four dynamic phenotypes | 0.992 | kappa |
| Development median baseline FI-Lab in matched-domain analysis | 0.300 | FI-Lab |

*All 2,930 dynamically evaluable patients were jointly classifiable. The Pearson correlation between primary and matched-domain change was 0.995; phenotype agreement was 99.5%; Cohen kappa was 0.992.*

**Supplementary Table S11C. Cross-classification of primary and matched-domain dynamic phenotypes.**

| **Primary phenotype** | **Matched: low-load stable** | **Matched: low-load worsened** | **Matched: high-load improved** | **Matched: high-load persistent** | **Total** |
| --- | --- | --- | --- | --- | --- |
| Low-load stable | 930 | 0 | 2 | 0 | 932 |
| Low-load worsened | 3 | 203 | 0 | 1 | 207 |
| High-load improved | 4 | 0 | 354 | 1 | 359 |
| High-load persistent | 0 | 2 | 2 | 1,428 | 1,432 |
| Total | 937 | 205 | 358 | 1,430 | 2,930 |

*Rows are primary classifications and columns are matched-domain classifications. Marginal totals refer to dynamically evaluable patients before the clinical complete-case exclusion.*

**Supplementary Table S12. Additional sensitivity analyses.**

| **analysis** | **development_n** | **development_events** | **validation_n** | **validation_events** | **model_converged** | **predictions_valid** | **validation_auc** | **validation_brier** |
| --- | --- | --- | --- | --- | --- | --- | --- | --- |
| 24 h Model B without CKD and diabetes covariates | 1,890 | 559 | 1,296 | 339 | Yes | Yes | 0.810 | 0.151 |
| 24 h Model B among patients without mechanical ventilation | 1,151 | 252 | 893 | 172 | Yes | Yes | 0.848 | 0.116 |

**Supplementary Table S13. Post-landmark mortality by phenotype and temporal cohort.**

| **split_temporal** | **dynamic_phenotype** | **n** | **events** | **event_rate** | **CI_low** | **CI_high** | **split_label** | **phenotype_label** | **y_base** | **y_plot** | **annotation** |
| --- | --- | --- | --- | --- | --- | --- | --- | --- | --- | --- | --- |
| development | High-load persistent | 916 | 339 | 0.370 | 0.339 | 0.402 | Development | High-load persistent | 1 | 1.140 | 37.0% (339/916) |
| temporal_validation | High-load improved | 137 | 43 | 0.314 | 0.242 | 0.396 | Temporal validation | High-load improved | 2 | 1.860 | 31.4% (43/137) |
| temporal_validation | High-load persistent | 508 | 190 | 0.374 | 0.333 | 0.417 | Temporal validation | High-load persistent | 1 | 0.860 | 37.4% (190/508) |
| development | Low-load stable | 499 | 45 | 0.090 | 0.068 | 0.119 | Development | Low-load stable | 4 | 4.140 | 9.0% (45/499) |
| temporal_validation | Low-load stable | 432 | 41 | 0.095 | 0.071 | 0.126 | Temporal validation | Low-load stable | 4 | 3.860 | 9.5% (41/432) |
| development | Low-load worsened | 120 | 31 | 0.258 | 0.188 | 0.343 | Development | Low-load worsened | 3 | 3.140 | 25.8% (31/120) |
| temporal_validation | Low-load worsened | 87 | 13 | 0.149 | 0.089 | 0.239 | Temporal validation | Low-load worsened | 3 | 2.860 | 14.9% (13/87) |
| development | High-load improved | 219 | 58 | 0.265 | 0.211 | 0.327 | Development | High-load improved | 2 | 2.140 | 26.5% (58/219) |

*Wilson 95% confidence intervals are supplied in the source table.*

**Supplementary Table S14. Decision-curve threshold ranges.**

| **landmark** | **dataset** | **model** | **interval** | **threshold_low** | **threshold_high** | **points** |
| --- | --- | --- | --- | --- | --- | --- |
| 24 h | temporal_validation | Model_A | 1 | 0.050 | 0.500 | 46 |
| 24 h | temporal_validation | Model_B | 1 | 0.050 | 0.500 | 46 |
| 72 h | temporal_validation | Model_A | 1 | 0.050 | 0.500 | 46 |
| 72 h | temporal_validation | Model_B | 1 | 0.050 | 0.500 | 46 |
| 72 h | temporal_validation | Model_C | 1 | 0.050 | 0.500 | 46 |
| 72 h | temporal_validation | Model_D | 1 | 0.050 | 0.500 | 46 |
| 72 h | temporal_validation | Model_E | 1 | 0.050 | 0.500 | 46 |

*All model curves are plotted with treat-all and treat-none strategies in Figure 3. No treatment threshold is recommended.*

**Supplementary Table S15. Sensitivity analyses restricted by early discharge.**

| **restriction** | **model** | **development_n** | **development_events** | **validation_n** | **validation_events** | **model_converged** | **predictions_valid** | **event_rate** | **brier** | **mean_predicted_risk** | **auc** | **auc_low** | **auc_high** | **calibration_intercept** | **calibration_slope** |
| --- | --- | --- | --- | --- | --- | --- | --- | --- | --- | --- | --- | --- | --- | --- | --- |
| Not hospital-discharged before 72 h | Model_A | 1,605 | 465 | 1,037 | 279 | Yes | Yes | 0.269 | 0.175 | 0.249 | 0.727 | 0.695 | 0.759 | 0.125 | 0.886 |
| Not hospital-discharged before 72 h | Model_B | 1,605 | 465 | 1,037 | 279 | Yes | Yes | 0.269 | 0.164 | 0.252 | 0.772 | 0.742 | 0.802 | 0.113 | 0.932 |
| Not hospital-discharged before 72 h | Model_D | 1,605 | 465 | 1,037 | 279 | Yes | Yes | 0.269 | 0.171 | 0.245 | 0.744 | 0.713 | 0.775 | 0.152 | 0.886 |
| Not hospital-discharged before 72 h | Model_E | 1,605 | 465 | 1,037 | 279 | Yes | Yes | 0.269 | 0.162 | 0.255 | 0.778 | 0.748 | 0.808 | 0.095 | 0.902 |
| Not ICU-discharged before 72 h | Model_A | 884 | 335 | 714 | 215 | Yes | Yes | 0.301 | 0.188 | 0.324 | 0.717 | 0.679 | 0.755 | -0.125 | 0.917 |
| Not ICU-discharged before 72 h | Model_B | 884 | 335 | 714 | 215 | Yes | Yes | 0.301 | 0.176 | 0.322 | 0.763 | 0.727 | 0.798 | -0.119 | 1.004 |
| Not ICU-discharged before 72 h | Model_D | 884 | 335 | 714 | 215 | Yes | Yes | 0.301 | 0.185 | 0.315 | 0.730 | 0.693 | 0.767 | -0.078 | 0.910 |
| Not ICU-discharged before 72 h | Model_E | 884 | 335 | 714 | 215 | Yes | Yes | 0.301 | 0.174 | 0.320 | 0.769 | 0.734 | 0.804 | -0.110 | 0.992 |

*Models were re-estimated after restricting to patients not discharged from hospital or ICU before 72 h.*

**Supplementary Table S16. Missing proportions for laboratory and model variables.**

| **cohort** | **variable** | **n** | **missing_n** | **missing_pct** |
| --- | --- | --- | --- | --- |
| full cohort | b_wbc | 3,376 | 163 | 0.048 |
| full cohort | b_hemoglobin | 3,376 | 161 | 0.048 |
| full cohort | b_platelet | 3,376 | 164 | 0.049 |
| full cohort | b_rdw | 3,376 | 163 | 0.048 |
| full cohort | b_creatinine | 3,376 | 147 | 0.044 |
| full cohort | b_bun | 3,376 | 146 | 0.043 |
| full cohort | b_glucose | 3,376 | 143 | 0.042 |
| full cohort | b_sodium | 3,376 | 141 | 0.042 |
| full cohort | b_potassium | 3,376 | 144 | 0.043 |
| full cohort | b_bicarbonate | 3,376 | 135 | 0.040 |
| full cohort | f_wbc | 3,376 | 313 | 0.093 |
| full cohort | f_hemoglobin | 3,376 | 314 | 0.093 |
| full cohort | f_platelet | 3,376 | 314 | 0.093 |
| full cohort | f_rdw | 3,376 | 315 | 0.093 |
| full cohort | f_creatinine | 3,376 | 292 | 0.086 |
| full cohort | f_bun | 3,376 | 292 | 0.086 |
| full cohort | f_glucose | 3,376 | 294 | 0.087 |
| full cohort | f_sodium | 3,376 | 288 | 0.085 |
| full cohort | f_potassium | 3,376 | 291 | 0.086 |
| full cohort | f_bicarbonate | 3,376 | 286 | 0.085 |
| full cohort | age_years | 3,376 | 0 | 0.000 |
| full cohort | gender | 3,376 | 0 | 0.000 |
| full cohort | gcs_min_24h | 3,376 | 15 | 0.004 |
| full cohort | vasopressor_24h | 3,376 | 0 | 0.000 |
| full cohort | ventilation_24h | 3,376 | 0 | 0.000 |
| full cohort | comorb_hypertension | 3,376 | 0 | 0.000 |
| full cohort | comorb_diabetes | 3,376 | 0 | 0.000 |
| full cohort | comorb_atrial_fibrillation | 3,376 | 0 | 0.000 |
| full cohort | comorb_ihd | 3,376 | 0 | 0.000 |
| full cohort | comorb_heart_failure | 3,376 | 0 | 0.000 |
| full cohort | comorb_ckd | 3,376 | 0 | 0.000 |
| full cohort | comorb_copd | 3,376 | 0 | 0.000 |
| full cohort | comorb_malignancy | 3,376 | 0 | 0.000 |
| 24 h baseline pool | b_wbc | 3,198 | 23 | 0.007 |
| 24 h baseline pool | b_hemoglobin | 3,198 | 22 | 0.007 |
| 24 h baseline pool | b_platelet | 3,198 | 23 | 0.007 |
| 24 h baseline pool | b_rdw | 3,198 | 23 | 0.007 |
| 24 h baseline pool | b_creatinine | 3,198 | 2 | 0.001 |
| 24 h baseline pool | b_bun | 3,198 | 1 | 0.000 |
| 24 h baseline pool | b_glucose | 3,198 | 2 | 0.001 |
| 24 h baseline pool | b_sodium | 3,198 | 0 | 0.000 |
| 24 h baseline pool | b_potassium | 3,198 | 2 | 0.001 |
| 24 h baseline pool | b_bicarbonate | 3,198 | 0 | 0.000 |
| 24 h baseline pool | f_wbc | 3,198 | 235 | 0.073 |
| 24 h baseline pool | f_hemoglobin | 3,198 | 236 | 0.074 |
| 24 h baseline pool | f_platelet | 3,198 | 236 | 0.074 |
| 24 h baseline pool | f_rdw | 3,198 | 237 | 0.074 |
| 24 h baseline pool | f_creatinine | 3,198 | 215 | 0.067 |
| 24 h baseline pool | f_bun | 3,198 | 215 | 0.067 |
| 24 h baseline pool | f_glucose | 3,198 | 216 | 0.068 |
| 24 h baseline pool | f_sodium | 3,198 | 211 | 0.066 |
| 24 h baseline pool | f_potassium | 3,198 | 214 | 0.067 |
| 24 h baseline pool | f_bicarbonate | 3,198 | 208 | 0.065 |
| 24 h baseline pool | age_years | 3,198 | 0 | 0.000 |
| 24 h baseline pool | gender | 3,198 | 0 | 0.000 |
| 24 h baseline pool | gcs_min_24h | 3,198 | 12 | 0.004 |
| 24 h baseline pool | vasopressor_24h | 3,198 | 0 | 0.000 |
| 24 h baseline pool | ventilation_24h | 3,198 | 0 | 0.000 |
| 24 h baseline pool | comorb_hypertension | 3,198 | 0 | 0.000 |
| 24 h baseline pool | comorb_diabetes | 3,198 | 0 | 0.000 |
| 24 h baseline pool | comorb_atrial_fibrillation | 3,198 | 0 | 0.000 |
| 24 h baseline pool | comorb_ihd | 3,198 | 0 | 0.000 |
| 24 h baseline pool | comorb_heart_failure | 3,198 | 0 | 0.000 |
| 24 h baseline pool | comorb_ckd | 3,198 | 0 | 0.000 |
| 24 h baseline pool | comorb_copd | 3,198 | 0 | 0.000 |
| 24 h baseline pool | comorb_malignancy | 3,198 | 0 | 0.000 |
| 72 h dynamic pool | b_wbc | 2,930 | 19 | 0.006 |
| 72 h dynamic pool | b_hemoglobin | 2,930 | 18 | 0.006 |
| 72 h dynamic pool | b_platelet | 2,930 | 18 | 0.006 |
| 72 h dynamic pool | b_rdw | 2,930 | 19 | 0.006 |
| 72 h dynamic pool | b_creatinine | 2,930 | 2 | 0.001 |
| 72 h dynamic pool | b_bun | 2,930 | 1 | 0.000 |
| 72 h dynamic pool | b_glucose | 2,930 | 2 | 0.001 |
| 72 h dynamic pool | b_sodium | 2,930 | 0 | 0.000 |
| 72 h dynamic pool | b_potassium | 2,930 | 2 | 0.001 |
| 72 h dynamic pool | b_bicarbonate | 2,930 | 0 | 0.000 |
| 72 h dynamic pool | f_wbc | 2,930 | 31 | 0.011 |
| 72 h dynamic pool | f_hemoglobin | 2,930 | 32 | 0.011 |
| 72 h dynamic pool | f_platelet | 2,930 | 32 | 0.011 |
| 72 h dynamic pool | f_rdw | 2,930 | 33 | 0.011 |
| 72 h dynamic pool | f_creatinine | 2,930 | 2 | 0.001 |
| 72 h dynamic pool | f_bun | 2,930 | 2 | 0.001 |
| 72 h dynamic pool | f_glucose | 2,930 | 2 | 0.001 |
| 72 h dynamic pool | f_sodium | 2,930 | 0 | 0.000 |
| 72 h dynamic pool | f_potassium | 2,930 | 0 | 0.000 |
| 72 h dynamic pool | f_bicarbonate | 2,930 | 0 | 0.000 |
| 72 h dynamic pool | age_years | 2,930 | 0 | 0.000 |
| 72 h dynamic pool | gender | 2,930 | 0 | 0.000 |
| 72 h dynamic pool | gcs_min_24h | 2,930 | 12 | 0.004 |
| 72 h dynamic pool | vasopressor_24h | 2,930 | 0 | 0.000 |
| 72 h dynamic pool | ventilation_24h | 2,930 | 0 | 0.000 |
| 72 h dynamic pool | comorb_hypertension | 2,930 | 0 | 0.000 |
| 72 h dynamic pool | comorb_diabetes | 2,930 | 0 | 0.000 |
| 72 h dynamic pool | comorb_atrial_fibrillation | 2,930 | 0 | 0.000 |
| 72 h dynamic pool | comorb_ihd | 2,930 | 0 | 0.000 |
| 72 h dynamic pool | comorb_heart_failure | 2,930 | 0 | 0.000 |
| 72 h dynamic pool | comorb_ckd | 2,930 | 0 | 0.000 |
| 72 h dynamic pool | comorb_copd | 2,930 | 0 | 0.000 |
| 72 h dynamic pool | comorb_malignancy | 2,930 | 0 | 0.000 |

*The full cohort, 24-hour baseline pool, and 72-hour dynamic pool are reported separately. No imputation was used.*

**Supplementary Table S17. FI-Lab laboratory missingness patterns.**

| **timepoint** | **split_temporal** | **pattern** | **N** |
| --- | --- | --- | --- |
| baseline 0-24 h | development | 1111111111 | 1902 |
| baseline 0-24 h | development | 0 | 60 |
| baseline 0-24 h | development | 111111 | 13 |
| baseline 0-24 h | development | 1 | 5 |
| baseline 0-24 h | development | 1111000000 | 2 |
| baseline 0-24 h | development | 1111011111 | 2 |
| baseline 0-24 h | development | 1111110111 | 2 |
| baseline 0-24 h | development | 1001 | 2 |
| baseline 0-24 h | development | 110 | 2 |
| baseline 0-24 h | development | 1111101111 | 1 |
| baseline 0-24 h | development | 100 | 1 |
| baseline 0-24 h | development | 11111 | 1 |
| baseline 0-24 h | development | 10 | 1 |
| baseline 0-24 h | development | 100111111 | 1 |
| baseline 0-24 h | development | 1111100000 | 1 |
| baseline 0-24 h | development | 10111111 | 1 |
| baseline 0-24 h | development | 1111111101 | 1 |
| baseline 0-24 h | temporal_validation | 1111111111 | 1294 |
| baseline 0-24 h | temporal_validation | 0 | 63 |
| baseline 0-24 h | temporal_validation | 111111 | 8 |
| baseline 0-24 h | temporal_validation | 1111000000 | 4 |
| baseline 0-24 h | temporal_validation | 1101111111 | 3 |
| baseline 0-24 h | temporal_validation | 100001001 | 1 |
| baseline 0-24 h | temporal_validation | 1 | 1 |
| baseline 0-24 h | temporal_validation | 10111111 | 1 |
| baseline 0-24 h | temporal_validation | 1001 | 1 |
| baseline 0-24 h | temporal_validation | 1111111101 | 1 |
| baseline 0-24 h | temporal_validation | 100 | 1 |
| follow-up 24-72 h | development | 1111111111 | 1830 |
| follow-up 24-72 h | development | 0 | 135 |
| follow-up 24-72 h | development | 111111 | 16 |
| follow-up 24-72 h | development | 1111000000 | 5 |
| follow-up 24-72 h | development | 1 | 3 |
| follow-up 24-72 h | development | 1111011111 | 2 |
| follow-up 24-72 h | development | 1111101111 | 1 |
| follow-up 24-72 h | development | 100 | 1 |
| follow-up 24-72 h | development | 1111000001 | 1 |
| follow-up 24-72 h | development | 1000111111 | 1 |
| follow-up 24-72 h | development | 110000 | 1 |
| follow-up 24-72 h | development | 1111110110 | 1 |
| follow-up 24-72 h | development | 1110111111 | 1 |
| follow-up 24-72 h | temporal_validation | 1111111111 | 1213 |
| follow-up 24-72 h | temporal_validation | 0 | 137 |
| follow-up 24-72 h | temporal_validation | 111111 | 17 |
| follow-up 24-72 h | temporal_validation | 1111000000 | 4 |
| follow-up 24-72 h | temporal_validation | 1 | 2 |
| follow-up 24-72 h | temporal_validation | 1111110111 | 2 |
| follow-up 24-72 h | temporal_validation | 100 | 1 |
| follow-up 24-72 h | temporal_validation | 1111000100 | 1 |
| follow-up 24-72 h | temporal_validation | 1111101111 | 1 |

*Pattern strings are ordered across the ten FI-Lab domains; 1 denotes observed and 0 denotes missing. The blank-looking zero pattern is retained as '0' in the source CSV.*

**Supplementary Table S18. Pre-landmark deaths and early discharge.**

| **interval** | **deaths_best_available** | **deaths_exact_timestamp** | **deaths_date_only** | **icu_discharges** | **hospital_discharges** |
| --- | --- | --- | --- | --- | --- |
| 0-24 h | 39 | 38 | 1 | 382 | 63 |
| 0-72 h | 148 | 134 | 14 | 1703 | 559 |

*Best-available timing used exact deathtime when available. Otherwise, a date-only death on or before the landmark calendar date was classified as pre-landmark. There was one date-only pre-landmark death at 24 h and 14 at 72 h. The alternative classification and its model results are reported in Supplementary Table S20.*

**Supplementary Table S19. Extracted MIMIC-IV item identifiers.**

| **source_variable** | **itemid** |
| --- | --- |
| b_bicarbonate_itemid | 50803 |
| b_bicarbonate_itemid | 50804 |
| b_bicarbonate_itemid | 50882 |
| b_bun_itemid | 51006 |
| b_creatinine_itemid | 50912 |
| b_glucose_itemid | 50809 |
| b_glucose_itemid | 50931 |
| b_hemoglobin_itemid | 50811 |
| b_hemoglobin_itemid | 51222 |
| b_platelet_itemid | 51265 |
| b_potassium_itemid | 50971 |
| b_rdw_itemid | 51277 |
| b_sodium_itemid | 50983 |
| b_wbc_itemid | 51301 |
| f_bicarbonate_itemid | 50803 |
| f_bicarbonate_itemid | 50804 |
| f_bicarbonate_itemid | 50882 |
| f_bun_itemid | 51006 |
| f_creatinine_itemid | 50912 |
| f_glucose_itemid | 50809 |
| f_glucose_itemid | 50931 |
| f_hemoglobin_itemid | 50811 |
| f_hemoglobin_itemid | 51222 |
| f_platelet_itemid | 51265 |
| f_potassium_itemid | 50971 |
| f_rdw_itemid | 51277 |
| f_sodium_itemid | 50983 |
| f_wbc_itemid | 51300 |
| f_wbc_itemid | 51301 |
| gcs_itemids_24h | 220739 |
| gcs_itemids_24h | 223900 |
| gcs_itemids_24h | 223901 |
| vasopressor_itemids_24h | 221289 |
| vasopressor_itemids_24h | 221653 |
| vasopressor_itemids_24h | 221662 |
| vasopressor_itemids_24h | 221749 |
| vasopressor_itemids_24h | 221906 |
| vasopressor_itemids_24h | 222315 |
| vasopressor_itemids_24h | 229630 |
| vasopressor_itemids_24h | 229632 |
| ventilation_itemids_24h | 220339 |
| ventilation_itemids_24h | 223848 |
| ventilation_itemids_24h | 223849 |
| ventilation_itemids_24h | 224684 |
| ventilation_itemids_24h | 224685 |
| ventilation_itemids_24h | 224686 |
| ventilation_itemids_24h | 224700 |
| ventilation_itemids_24h | 227565 |
| ventilation_itemids_24h | 227566 |
| ventilation_itemids_24h | 229314 |

*The SQL additionally resolves labels and source tables; this table records the item identifiers present in the final extracted dataset.*

**Supplementary Table S20. Sensitivity analysis for date-only deaths at the 24-hour and 72-hour landmarks.**

| **Landmark** | **Scenario** | **Date-only deaths** | **Entering analysis** | **Development events/n** | **Validation events/n** | **Validation AUC (95% CI)** | **Brier** | **Calibration intercept / slope** |
| --- | --- | --- | --- | --- | --- | --- | --- | --- |
| 24 h | excluded from landmark risk set | 1 | 0 | 559/1,890 | 339/1,296 | 0.808 (0.784-0.833) | 0.152 | 0.095 / 0.943 |
| 24 h | treated as alive at landmark | 1 | 1 | 560/1,891 | 339/1,296 | 0.809 (0.784-0.833) | 0.152 | 0.094 / 0.942 |
| 72 h | excluded from landmark risk set | 14 | 0 | 473/1,754 | 287/1,164 | 0.798 (0.770-0.825) | 0.150 | 0.104 / 0.906 |
| 72 h | treated as alive at landmark | 14 | 2 | 474/1,755 | 288/1,165 | 0.798 (0.771-0.825) | 0.150 | 0.107 / 0.906 |

*Primary analyses classified date-only deaths on or before the landmark calendar date as pre-landmark and excluded them from the corresponding risk set. The alternative scenario treated all date-only deaths as alive at the landmark. Only records meeting the remaining evaluability and complete-case criteria entered the alternative model cohorts.*

**Supplementary Table S21. Baseline comparison between development and temporal-validation cohorts.**

| **Cohort** | **Characteristic** | **Development** | **Temporal validation** | **SMD** |
| --- | --- | --- | --- | --- |
| Full admission cohort | Age, years | 70.228 (15.367) | 68.164 (15.298) | -0.135 |
| Full admission cohort | Male sex | 974/1998 (48.7%) | 731/1378 (53.0%) | +0.086 |
| Full admission cohort | Minimum GCS in first 24 h | 9.856 (4.605) | 10.761 (4.505) | +0.199 |
| Full admission cohort | Vasopressor use in first 24 h | 481/1998 (24.1%) | 246/1378 (17.9%) | -0.153 |
| Full admission cohort | Mechanical ventilation in first 24 h | 768/1998 (38.4%) | 420/1378 (30.5%) | -0.168 |
| Full admission cohort | Hypertension | 1536/1998 (76.9%) | 1012/1378 (73.4%) | -0.080 |
| Full admission cohort | Diabetes | 675/1998 (33.8%) | 418/1378 (30.3%) | -0.074 |
| Full admission cohort | Atrial fibrillation | 779/1998 (39.0%) | 489/1378 (35.5%) | -0.073 |
| Full admission cohort | Ischemic heart disease | 699/1998 (35.0%) | 394/1378 (28.6%) | -0.138 |
| Full admission cohort | Heart failure | 548/1998 (27.4%) | 300/1378 (21.8%) | -0.132 |
| Full admission cohort | Chronic kidney disease | 472/1998 (23.6%) | 209/1378 (15.2%) | -0.215 |
| Full admission cohort | COPD | 234/1998 (11.7%) | 125/1378 (9.1%) | -0.087 |
| Full admission cohort | Malignancy | 275/1998 (13.8%) | 170/1378 (12.3%) | -0.042 |
| Full admission cohort | Baseline FI-Lab | 0.349 (0.209) | 0.312 (0.219) | -0.175 |
| Full admission cohort | AIS as principal diagnosis | 1089/1998 (54.5%) | 875/1378 (63.5%) | +0.184 |
| 24 h complete-case analysis cohort | Age, years | 70.128 (15.282) | 67.999 (15.268) | -0.139 |
| 24 h complete-case analysis cohort | Male sex | 921/1890 (48.7%) | 689/1296 (53.2%) | +0.089 |
| 24 h complete-case analysis cohort | Minimum GCS in first 24 h | 9.832 (4.602) | 10.712 (4.517) | +0.193 |
| 24 h complete-case analysis cohort | Vasopressor use in first 24 h | 465/1890 (24.6%) | 239/1296 (18.4%) | -0.150 |
| 24 h complete-case analysis cohort | Mechanical ventilation in first 24 h | 739/1890 (39.1%) | 403/1296 (31.1%) | -0.168 |
| 24 h complete-case analysis cohort | Hypertension | 1452/1890 (76.8%) | 953/1296 (73.5%) | -0.076 |
| 24 h complete-case analysis cohort | Diabetes | 635/1890 (33.6%) | 396/1296 (30.6%) | -0.065 |
| 24 h complete-case analysis cohort | Atrial fibrillation | 739/1890 (39.1%) | 462/1296 (35.6%) | -0.071 |
| 24 h complete-case analysis cohort | Ischemic heart disease | 660/1890 (34.9%) | 369/1296 (28.5%) | -0.139 |
| 24 h complete-case analysis cohort | Heart failure | 520/1890 (27.5%) | 287/1296 (22.1%) | -0.124 |
| 24 h complete-case analysis cohort | Chronic kidney disease | 447/1890 (23.7%) | 201/1296 (15.5%) | -0.206 |
| 24 h complete-case analysis cohort | COPD | 222/1890 (11.7%) | 121/1296 (9.3%) | -0.079 |
| 24 h complete-case analysis cohort | Malignancy | 261/1890 (13.8%) | 157/1296 (12.1%) | -0.050 |
| 24 h complete-case analysis cohort | Baseline FI-Lab | 0.346 (0.206) | 0.308 (0.216) | -0.181 |
| 24 h complete-case analysis cohort | AIS as principal diagnosis | 1018/1890 (53.9%) | 809/1296 (62.4%) | +0.174 |
| 72 h complete-case analysis cohort | Age, years | 69.714 (15.296) | 67.921 (15.102) | -0.118 |
| 72 h complete-case analysis cohort | Male sex | 870/1754 (49.6%) | 624/1164 (53.6%) | +0.080 |
| 72 h complete-case analysis cohort | Minimum GCS in first 24 h | 9.849 (4.603) | 10.653 (4.509) | +0.176 |
| 72 h complete-case analysis cohort | Vasopressor use in first 24 h | 443/1754 (25.3%) | 224/1164 (19.2%) | -0.145 |
| 72 h complete-case analysis cohort | Mechanical ventilation in first 24 h | 693/1754 (39.5%) | 373/1164 (32.0%) | -0.156 |
| 72 h complete-case analysis cohort | Hypertension | 1354/1754 (77.2%) | 863/1164 (74.1%) | -0.071 |
| 72 h complete-case analysis cohort | Diabetes | 599/1754 (34.2%) | 367/1164 (31.5%) | -0.056 |
| 72 h complete-case analysis cohort | Atrial fibrillation | 681/1754 (38.8%) | 418/1164 (35.9%) | -0.060 |
| 72 h complete-case analysis cohort | Ischemic heart disease | 616/1754 (35.1%) | 339/1164 (29.1%) | -0.129 |
| 72 h complete-case analysis cohort | Heart failure | 487/1754 (27.8%) | 269/1164 (23.1%) | -0.107 |
| 72 h complete-case analysis cohort | Chronic kidney disease | 423/1754 (24.1%) | 184/1164 (15.8%) | -0.209 |
| 72 h complete-case analysis cohort | COPD | 203/1754 (11.6%) | 108/1164 (9.3%) | -0.075 |
| 72 h complete-case analysis cohort | Malignancy | 239/1754 (13.6%) | 137/1164 (11.8%) | -0.056 |
| 72 h complete-case analysis cohort | Baseline FI-Lab | 0.348 (0.205) | 0.315 (0.213) | -0.155 |
| 72 h complete-case analysis cohort | AIS as principal diagnosis | 906/1754 (51.7%) | 696/1164 (59.8%) | +0.164 |

*Continuous variables are mean (SD); binary variables are n/N (%). SMD is signed as temporal validation minus development. Missing counts were 12 and 3 for GCS and 60 and 63 for baseline FI-Lab in the full development and validation cohorts, respectively; complete-case landmark cohorts had no missing values in listed model variables.*

**Supplementary Table S22A. Frequency and 90-day mortality of principal-diagnosis AIS.**

| **Cohort** | **Total n** | **Principal AIS, n (%)** | **Nonprincipal AIS, n (%)** | **Principal AIS deaths/n (%)** | **Nonprincipal AIS deaths/n (%)** |
| --- | --- | --- | --- | --- | --- |
| Overall | 3,376 | 1,964 (58.2%) | 1,412 (41.8%) | 457/1,964 (23.3%) | 519/1,412 (36.8%) |
| Development | 1,998 | 1,089 (54.5%) | 909 (45.5%) | 286/1,089 (26.3%) | 325/909 (35.8%) |
| Temporal validation | 1,378 | 875 (63.5%) | 503 (36.5%) | 171/875 (19.5%) | 194/503 (38.6%) |

*Principal-diagnosis AIS required a qualifying AIS code at diagnosis sequence position 1. Mortality in this table is all-cause death through day 90 from ICU admission.*

**Supplementary Table S22B. Landmark-model sensitivity analysis restricted to principal-diagnosis AIS.**

| **Landmark** | **Cohort** | **Model** | **Events/n** | **AUC (95% CI)** | **Brier** | **Calibration intercept** | **Calibration slope** |
| --- | --- | --- | --- | --- | --- | --- | --- |
| 24 h | Development | Model A | 258/1,018 | 0.861 (0.835-0.887) | 0.125 | 0.000 | 1.000 |
| 24 h | Temporal validation | Model A | 154/809 | 0.852 (0.820-0.884) | 0.115 | 0.063 | 0.862 |
| 24 h | Development | Model B | 258/1,018 | 0.866 (0.841-0.891) | 0.123 | 0.000 | 1.000 |
| 24 h | Temporal validation | Model B | 154/809 | 0.872 (0.843-0.901) | 0.109 | 0.041 | 0.898 |
| 72 h | Development | Model A | 192/906 | 0.840 (0.808-0.871) | 0.122 | 0.000 | 1.000 |
| 72 h | Temporal validation | Model A | 116/696 | 0.815 (0.776-0.855) | 0.118 | 0.111 | 0.809 |
| 72 h | Development | Model B | 192/906 | 0.844 (0.813-0.874) | 0.121 | 0.000 | 1.000 |
| 72 h | Temporal validation | Model B | 116/696 | 0.837 (0.801-0.874) | 0.112 | 0.086 | 0.871 |
| 72 h | Development | Model C | 192/906 | 0.852 (0.823-0.882) | 0.117 | 0.000 | 1.000 |
| 72 h | Temporal validation | Model C | 116/696 | 0.838 (0.803-0.873) | 0.112 | 0.123 | 0.835 |
| 72 h | Development | Model D | 192/906 | 0.848 (0.818-0.878) | 0.118 | 0.000 | 1.000 |
| 72 h | Temporal validation | Model D | 116/696 | 0.821 (0.784-0.857) | 0.118 | 0.166 | 0.794 |
| 72 h | Development | Model E | 192/906 | 0.858 (0.829-0.887) | 0.116 | 0.000 | 1.000 |
| 72 h | Temporal validation | Model E | 116/696 | 0.835 (0.800-0.869) | 0.115 | 0.080 | 0.788 |

*Models were re-estimated in principal-diagnosis AIS development cohorts and transferred without refitting to the corresponding temporal-validation cohorts.*

**Supplementary Table S23. Restricted cubic spline tests of continuous FI-Lab functional form.**

| **Landmark** | **Analysis** | **Predictor** | **Events/n** | **LR chi-square (df)** | **P for nonlinearity** | **Evidence of nonlinearity** |
| --- | --- | --- | --- | --- | --- | --- |
| 24 h | baseline FI-Lab | Baseline FI-Lab (per 0.1) | 559/1,890 | 4.524 (3) | 0.210 | No |
| 72 h | baseline FI-Lab adjusted for change | Baseline FI-Lab (per 0.1) | 473/1,754 | 3.863 (3) | 0.277 | No |
| 72 h | FI-Lab change adjusted for baseline | FI-Lab change (per 0.1) | 473/1,754 | 4.575 (3) | 0.206 | No |

*Each natural cubic spline had restricted linear tails and 4 degrees of freedom. Likelihood-ratio tests compared the spline model with the corresponding linear model in development data; all models converged.*

**Supplementary Figure S1. Adjusted relationships of baseline FI-Lab and FI-Lab change with post-landmark mortality.**

*Curves show adjusted predicted mortality with 95% confidence intervals from natural cubic spline models. The 24-hour panel assesses baseline FI-Lab; the 72-hour panels assess baseline FI-Lab adjusted for change and change adjusted for baseline. Likelihood-ratio tests found no evidence of nonlinearity.*
